# Type I-like behavior of the type II α7 nicotinic acetylcholine receptor positive allosteric modulator A-867744

**DOI:** 10.7717/peerj.7542

**Published:** 2019-09-02

**Authors:** Krisztina Pesti, Peter Lukacs, Arpad Mike

**Affiliations:** 1 MTA-ELTE NAP B Opto-Neuropharmacology Group, Eötvös Loránd University, Budapest, Hungary; 2 Department of Biochemistry, Eötvös Loránd University, Budapest, Hungary; 3 School of PhD Studies, Semmelweis University, Budapest, Hungary; 4 Plant Protection Institute, Centre for Agricultural Research, Hungarian Academy of Sciences, Martonvásár, Hungary

**Keywords:** Positive allosteric modulator, PNU-120596, Choline, Cognitive enhancement, α7 nAChR, A-867744, Type II PAM, Patch clamp

## Abstract

Cognitive impairment often involves the decreased expression or hypofunction of alpha 7-type nicotinic acetylcholine receptors (α7 nAChRs). Agonists or positive allosteric modulators (PAMs) of α7 nAChRs are known to be potential treatments for dementias, different neurodegenerative disorders, pain syndromes and conditions involving inflammation. In some of these conditions, it is desirable to maintain the temporal precision of fast cholinergic events, while in others, this temporal precision is unnecessary. For this reason, the optimal therapeutic effect for distinct indications may require PAMs with different mechanisms of action. The two major mechanisms are called “type I”, which are compounds that augment α7 nAChR-mediated currents but maintain their characteristic fast kinetics; and “type II”, which are compounds that produce augmented and prolonged currents. In this study, we performed a kinetic analysis of two type II PAMs of the α7 nAChR: PNU-120596 and A-867744, using a fast perfusion method that allowed high temporal resolution. We characterized the type of modulation produced by the two compounds, the state-dependence of the modulatory action, and the interaction between the two compounds. We found fundamental differences between the modulation mechanisms by PNU-120596 and A-867744. Most importantly, during brief agonist pulses, A-867744 caused a strikingly type I-like modulation, while PNU-120596 caused a type II-like prolonged activation. Our results demonstrate that specific compounds, even though all labeled as type II PAMs, can behave in completely different ways, including their onset and offset kinetics, state preference, and single channel open time. Our results emphasize that subtle details of the mechanism of action may be significant in assessing the therapeutic applicability of α7 nAChR PAM compounds.

## Introduction

Nicotinic acetylcholine receptors are members of the cys-loop superfamily of ligand-gated ion channels (Faghih, Gfesser & Gopalakrishnan, 2007; Albuquerque et al., 2009). They are cation-selective membrane proteins with either hetero- or homopentameric structures. The neuronal type alpha 7 nicotinic acetylcholine receptors (α7 nAChR) are homopentameric, i.e., they are composed of five identical subunits. Alpha 7 nAChRs are highly expressed in the central nervous system (Zoli, Pistillo & Gotti, 2015). They have an important role in attentional function, memory processes, and cholinergic anti-inflammatory pathways (Thomsen et al., 2010; Kalkman & Feuerbach, 2016; Pavlov et al., 2003). Hypofunction of the α7 nAChRs contributes to cognitive symptoms in schizophrenia and Alzheimer’s disease (Wallace & Porter, 2011). Selective agonists and positive allosteric modulators (PAMs) are prospective drugs with pro-cognitive, anti-inflammatory, neuroprotective, and analgesic effects (Uteshev, 2014; Kalkman & Feuerbach, 2016; Hoover, 2017). Systemic application of agonists may cause activation but also desensitization of the receptors, i.e., it may not help but rather impede the response to physiological neuronal activity. In contrast, in the presence of PAMs, the physiological spatiotemporal pattern of activity is maintained, and responses are augmented. In this respect, one might suppose that type I PAMs (that only cause the augmentation of agonist-evoked currents but do not change their kinetics) (Grønlien et al., 2007b) should be better therapeutic agents than type II PAMs, which radically prolong the period of receptor activation. In addition, long-term agonist treatment-induced upregulation of α7 nAChRs has been shown to be inhibited by type II but not type I PAMs (Thomsen & Mikkelsen, 2012). It is important to note, however, that the type I vs. type II classification is a rather superficial oversimplification (Chatzidaki & Millar, 2015; Chatzidaki et al., 2015; Corradi & Bouzat, 2016), and type II PAMs do not form a homogenous group. For example, it was found that TQS (4-naphthalen-1-yl-3a,4,5,9b-tetrahydro-3H-cyclopenta[c]quinoline-8-sulfonamide) and PNU-120596 act differently from A-867744, as the former two compounds evoke two-component currents (Malysz et al., 2009). In the case of PNU-120596, we previously demonstrated that high temporal resolution, achieved by an ultrafast solution switching system (Pesti et al., 2014), can provide valuable insights into the mechanism of action of PAM molecules. We managed to identify the first component as being the unmodulated agonist-evoked response (Szabo et al., 2014). The fact that PNU-120596 was unable to alter the first component implied that this compound was ineffective against resting receptors and could only exert its effect after the agonist had induced a conformational transition. The aim of this study was to perform a similar detailed and high-temporal precision investigation for A-867744, which is a special kind of type II PAM (Newcombe et al., 2018) and to compare its properties with those of PNU-120596. We also intended to investigate possible interactions between the two compounds, i.e., whether we can observe displacement or cooperativity between them. A-867744 has been shown to displace the agonist [^3^H]A-585539 (Malysz et al., 2009), but this could not be attributed to competition for the orthosteric binding site because the antagonist [^3^H]MLA was not displaced. Furthermore, displacement was absent in chimeric receptors where the orthosteric binding site of the α7 nAChR was intact but the transmembrane regions were replaced by those of the 5-HT3 receptor. Indeed, in mutagenesis experiments, the binding sites for the type II PAMs PNU-120596 and A-867744, the binding site for the type I PAM NS-1738, and the binding site for compound PAM-2 (Arias et al., 2011) have all been found to share key residues (Collins, Young & Millar, 2011; Young et al., 2008; Marotta et al., 2015; Andersen et al., 2016; Newcombe et al., 2018). The area where the overlapping binding sites are located is an intrasubunit cavity within each transmembrane domain of the pentameric receptor. For potentiation by PNU-120596, four or five of the binding sites must be occupied (DaCosta & Sine, 2013). The stoichiometry of potentiation by A-867744 is thus far unknown.

## Materials & Methods

### Compounds

4-(5-(4-Chlorophenyl)-2-methyl-3-propionyl-1H-pyrrol-1-yl)benzenesulfonamide (A-867744) and [1-(5-Chloro-2,4-dimethoxyphenyl)-3-(5-methylisoxazol-3-yl)-urea] (PNU 120596) were purchased from Tocris Bioscience and Abcam, respectively. Choline and all other chemicals were purchased from Sigma-Aldrich. Modulators were dissolved in DMSO. Stock solutions (10 mM) were diluted to the appropriate concentration before the experiments. PNU-120596 and A-867744 were used at a 1 µM concentration unless otherwise noted, and choline was used at a 10 mM concentration. Both modulators are strongly lipophilic and could not be fully washed out by saline alone. For this reason, in one side of the theta-tube only one of the modulator compounds was used throughout the day. The washing protocol at the end of each day consisted of rinsing the perfusion system with a 1 to 10 mixture of diethyl ether and ethanol and then with 2-propanol, ethanol and water.

### Cell cultures

GH4C1 cells stably transfected with pCEP4/rat α7 nAChR were obtained from Siena Biotech S.p.A. (Siena, Italy). GH4C1 cells endogenously express the assembly promoting chaperone protein RIC-3 (Williams et al., 2005). Cells were cultured in poly-L-lysine (PLL)-coated standard tissue culture flasks (T-25) at 37 °C in 95% humidified air and 5% CO_2_. Culture medium contained Ham’s F10 Nutrient Mixture (Gibco), 15% horse serum (Gibco), 2.5% fetal bovine serum (Gibco), 1% penicillin–streptomycin (Lonza), 1 mM GlutaMAX (Gibco) and 100 µg/ml Hygromycin B (Invitrogen). Before the experiments, at ∼80% confluency, the cells were plated onto PLL-coated Petri dishes (*d* = 35 mm) for use on the following day.

### Electrophysiology

Experiments were performed in whole-cell or outside-out patch configurations using an Axopatch 200B amplifier and the pClamp software (Molecular Devices, Sunnyvale, CA). Currents were recorded at a holding potential of −70 mV, digitized at 20 kHz and filtered at 10 kHz. Borosilicate glass pipettes (World Precision Instruments) were pulled with a P-87 micropipette puller (Sutter Instruments) and filled with pipette solution (50 mM CsCl, 60 mM CsF, 10 mM NaCl, 10 mM HEPES, and 20 mM EGTA, pH 7.2). Pipette resistances ranged between 1.7 and 4.0 M Ω, and the series resistance values were between 2.1 and 9.1 M Ω. Experiments were carried out at room temperature (∼25 °C). Cells were transferred to the recording chamber, and the culture medium was exchanged to a HEPES-containing extracellular solution (140 mM NaCl, 5 mM KCl, 2 mM CaCl2, 1 mM MgCl2, 5 mM HEPES-Na, 10 mM D-glucose, pH adjusted to 7.3). The osmolarity values (∼330 mOsm) of the solutions were balanced with D-glucose.

### Perfusion protocols

**Figure 1:**
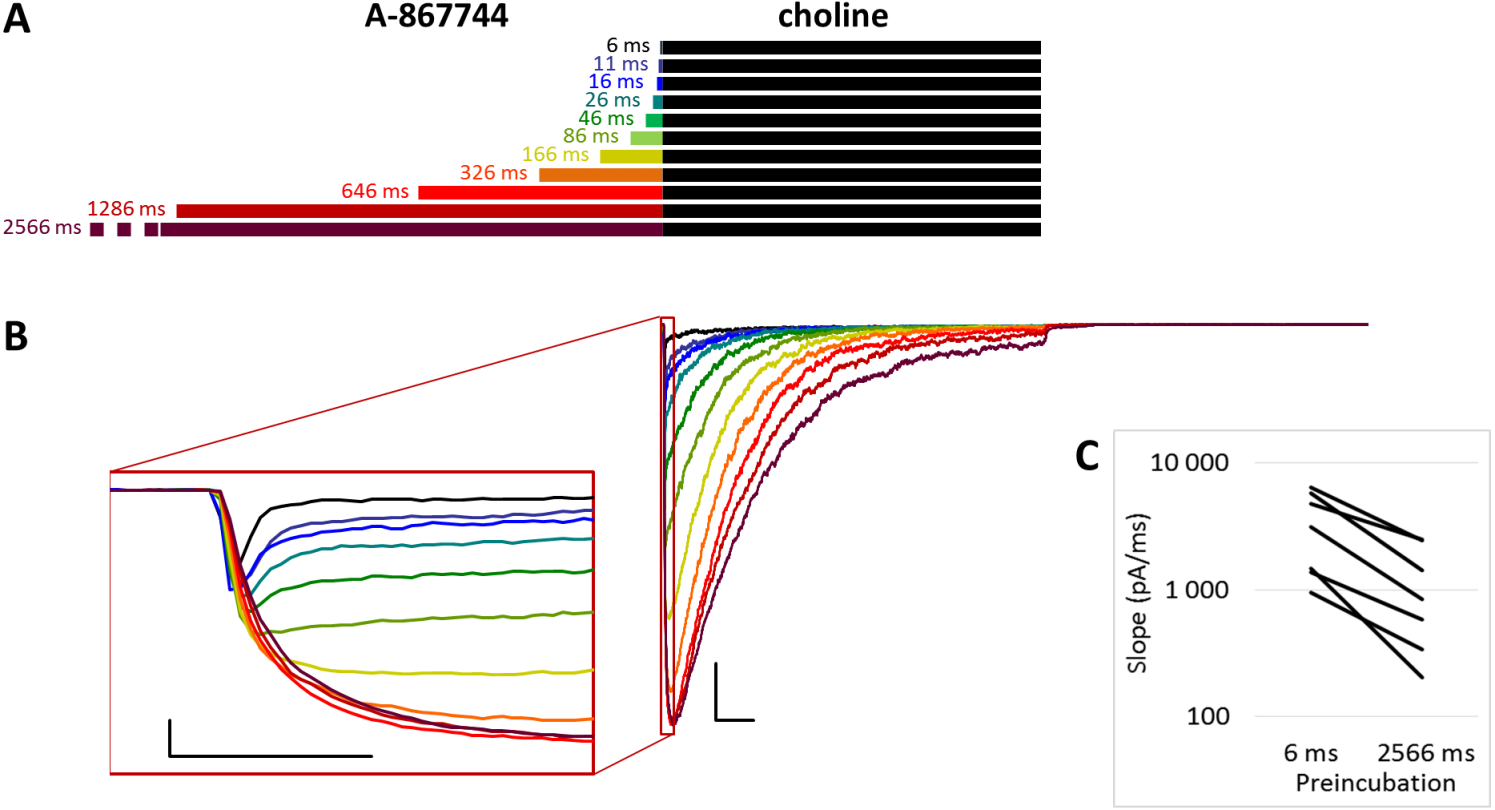
Effects of different lengths of A-867744 preincubation on choline-evoked currents. (A) Perfusion protocol with preincubation durations indicated. Colors in (B) indicate corresponding lengths of preincubation as shown in (A). (B) A typical example for the effect of 1 µM A-867744 preincubation on currents evoked by a 1 s pulse of 10 mM choline, and on the deactivation after it. Scale bars: 100 ms, 1 nA. Inset shows the first 20 ms of the agonist-evoked current on an expanded time scale. Scale bars: 10 ms, 1 nA. (C) The change in maximal slope values of the initial phase of the current, depending on the length of modulator preincubation. Data from *n* = 7 cells. Only data from the shortest (6 ms) and longest (2,566 ms) preincubation are shown.

**Figure 2:**
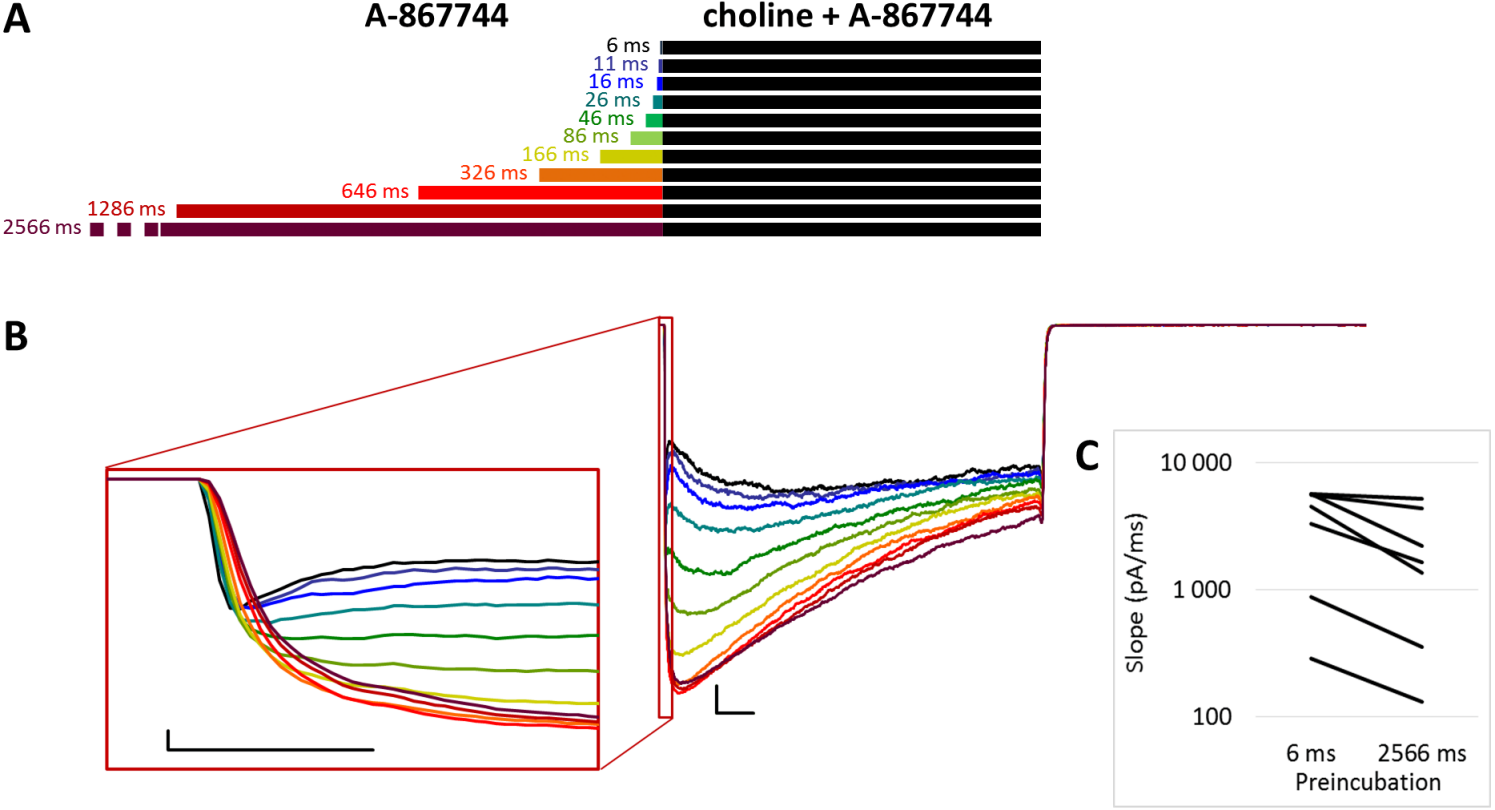
Effects of different lengths of A-867744 preincubation on currents evoked by coapplied choline and A-867744. (A) Perfusion protocol. (B) A typical example for the effect. Scale bars: 100 ms, 1 nA. Inset shows the first 20 ms on an expanded time scale. Scale bars: 10 ms, 1 nA. (C) The change in maximal slope values of the initial phase of the current, depending on the length of modulator preincubation. Only data from the shortest (6 ms) and longest (2,566 ms) preincubation are shown.

**Figure 3:**
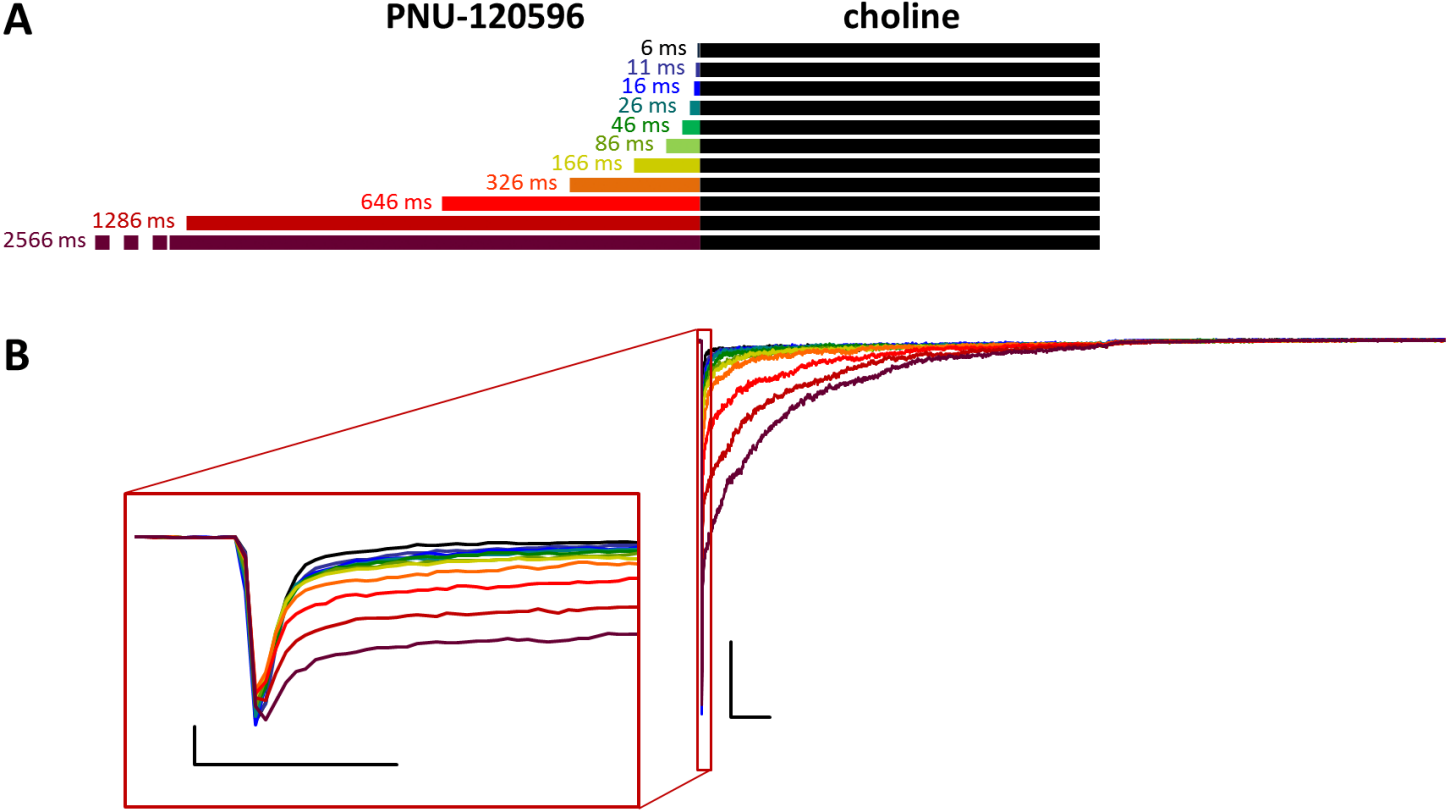
Effects of different lengths of PNU-120596 preincubation on choline evoked currents. (A) Perfusion protocol. (B) A typical example for the effect of 1 µM PNU-120596 preincubation on currents evoked by a 1 s pulse of 10 mM choline. Scale bars: 100 ms, 1 nA. Inset shows the first 20 ms of the agonist-evoked current at an expanded time scale. Scale bars: 10 ms, 1 nA.

**Figure 4:**
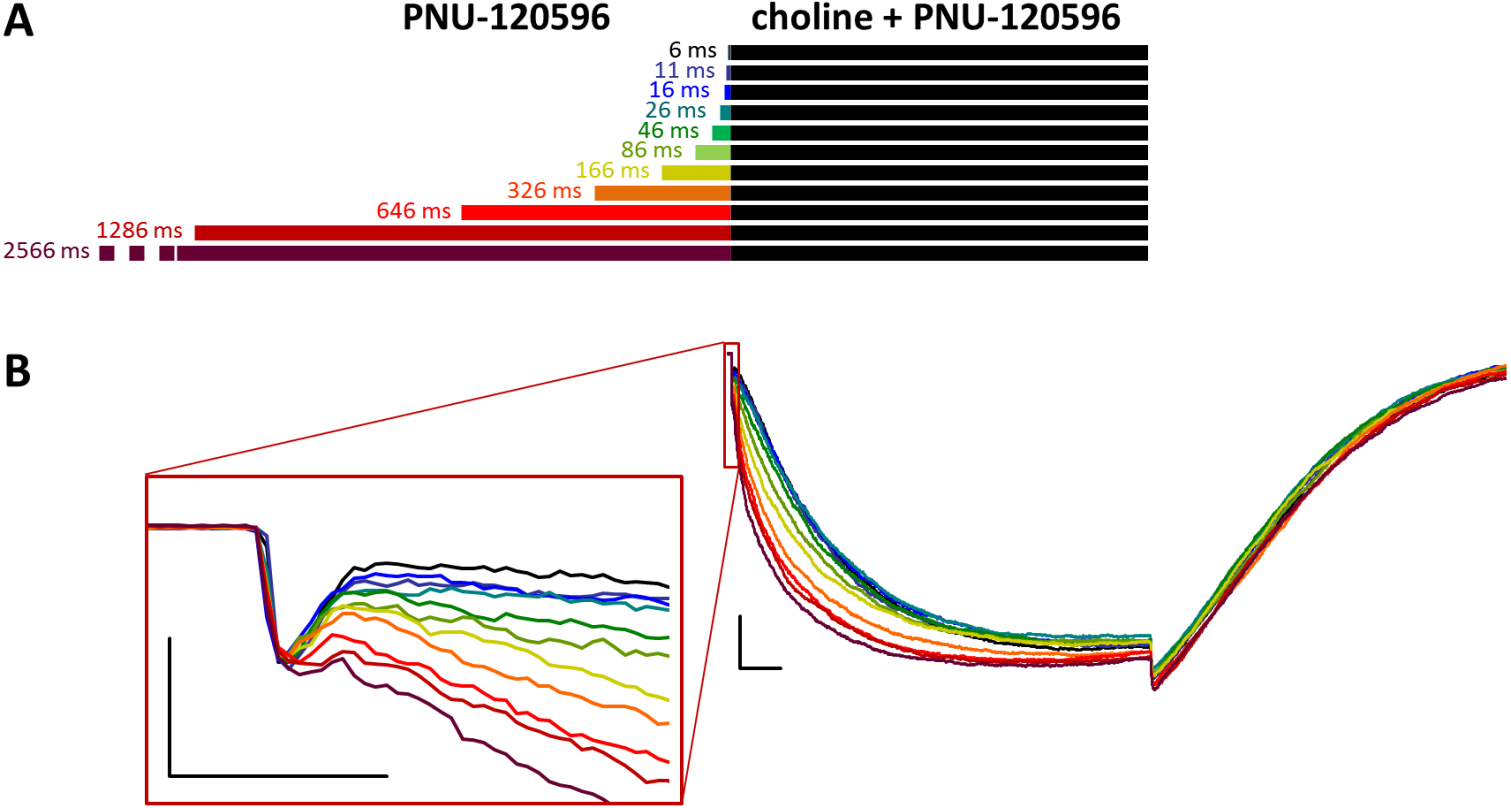
Effects of different lengths of PNU-120596 preincubation on currents evoked by coapplied choline and PNU-120596. (A) Perfusion protocol. (B) A typical example for the effect. Scale bars: 100 ms, 1 nA. Inset shows the first 20 ms on an expanded time scale. Scale bars: 10 ms, 1 nA.

**Figure 5:**
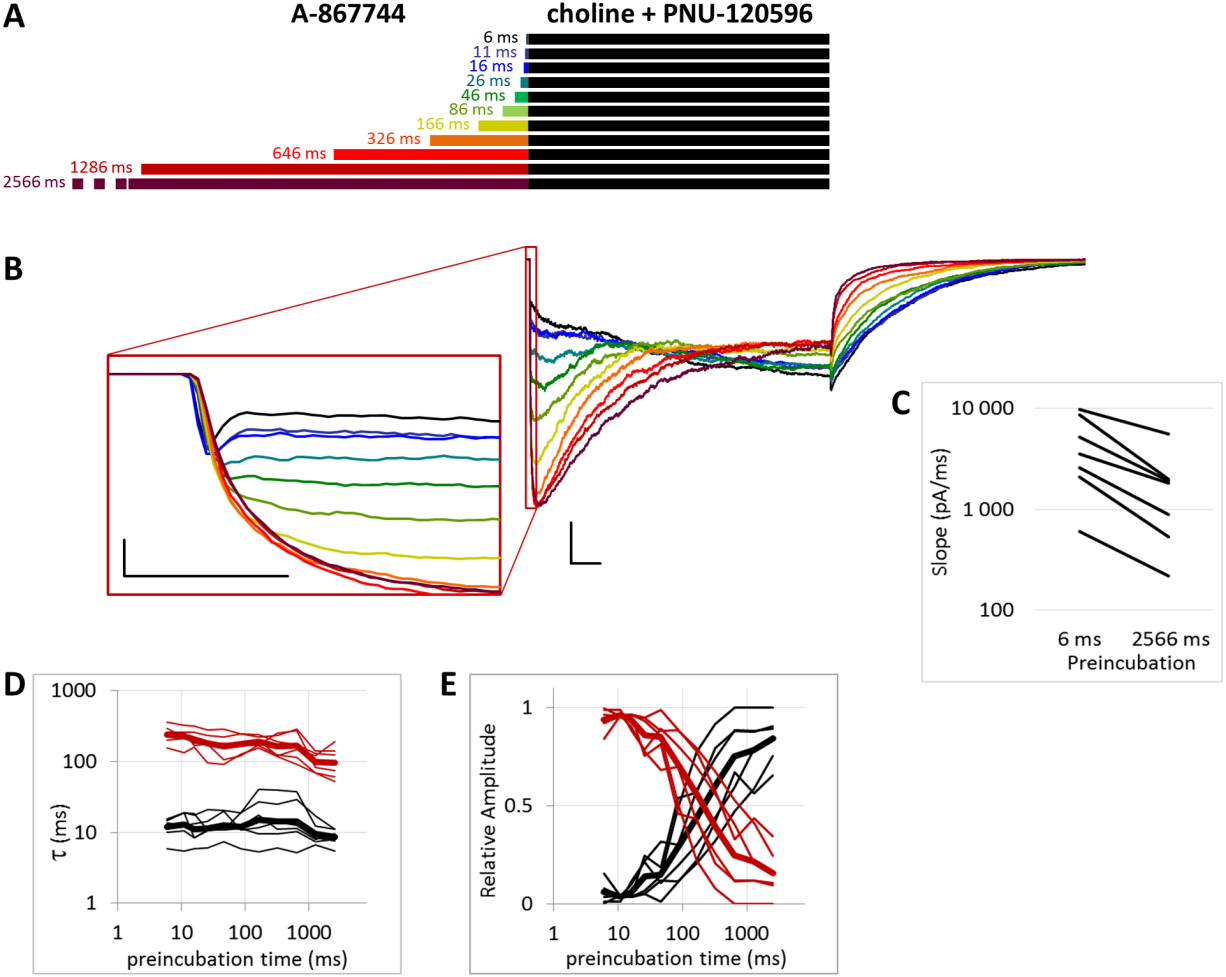
Effects of different lengths of 1 µM A-867744 preincubation on currents evoked by coapplied choline and 1 µM PNU-120596. (A) Perfusion protocol. (B) A typical example for the effect. Scale bars: 100 ms, 1 nA. Inset shows the first 20 ms on an expanded time scale. Scale bars: 10 ms, 1 nA. (C) The change in maximal slope values of the initial phase of the current, depending on the length of modulator preincubation. Only data from the shortest (6 ms) and longest (2,566 ms) preincubation are shown. (D) and (E) Fast (black) and slow (red) time constants of deactivation (D), and their respective contribution to the amplitude (E). Thin lines show data from 7 individual measurements, thick lines show geometric mean for time constants, and arithmetic mean for relative amplitudes.

**Figure 6:**
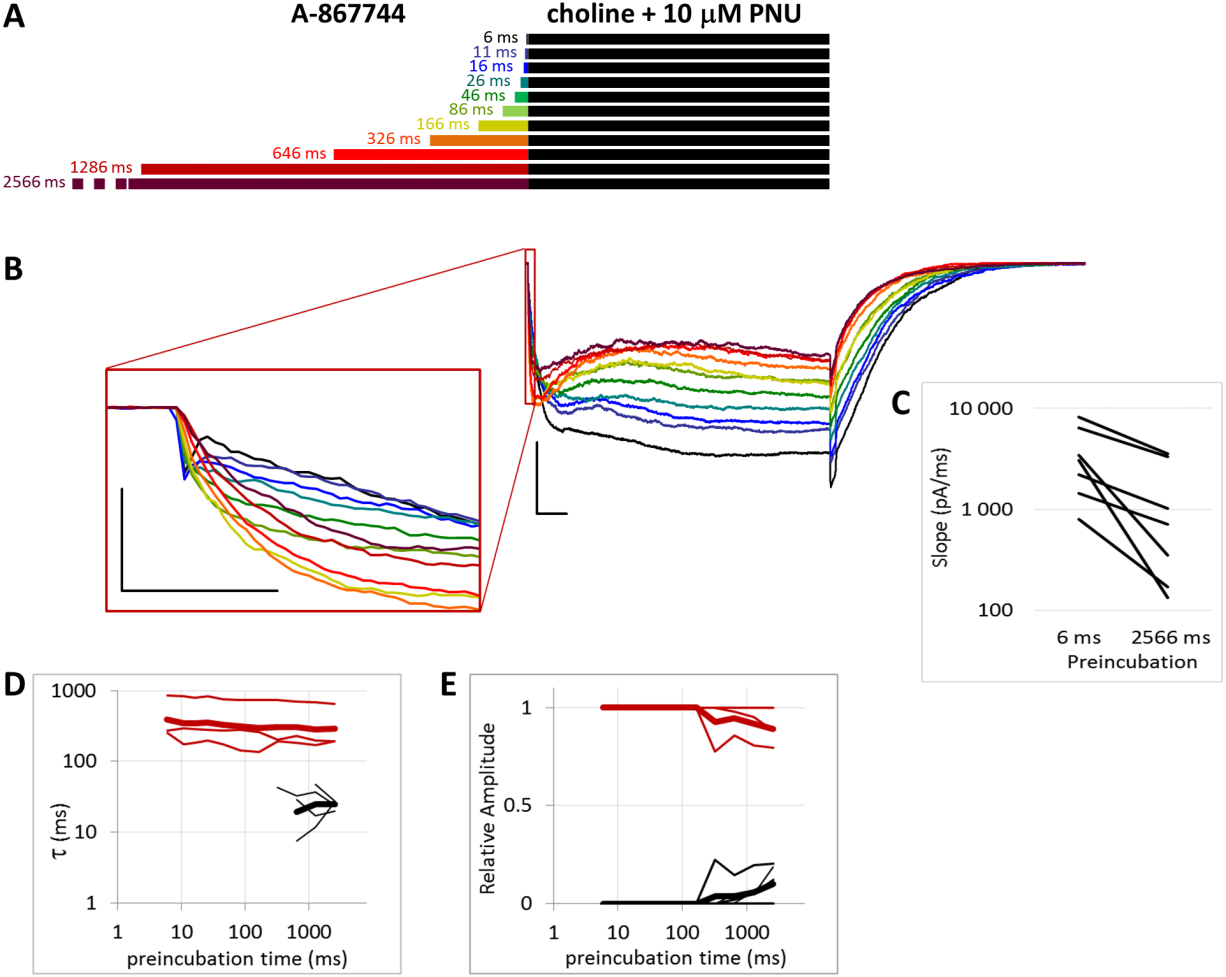
Effects of different lengths of 1 µM A-867744 preincubation on currents evoked by coapplied choline and 10 µM PNU-120596. (A) Perfusion protocol. (B) A typical example for the effect. Scale bars: 100 ms, 1 nA. Inset shows the first 20 ms of the agonist-evoked current at an expanded time scale. Scale bars: 10 ms, 1 nA. (C) The change in maximal slope values of the initial phase of the current, depending on the length of modulator preincubation. Only data from the shortest (6 ms) and longest (2,566 ms) preincubation are shown. (D) and (E) Fast (black) and slow (red) time constants of deactivation (D), and their respective contribution to the amplitude (E). Thin lines show data from six individual measurements, thick lines show geometric mean for time constants, and arithmetic mean for relative amplitudes.

**Figure 7:**
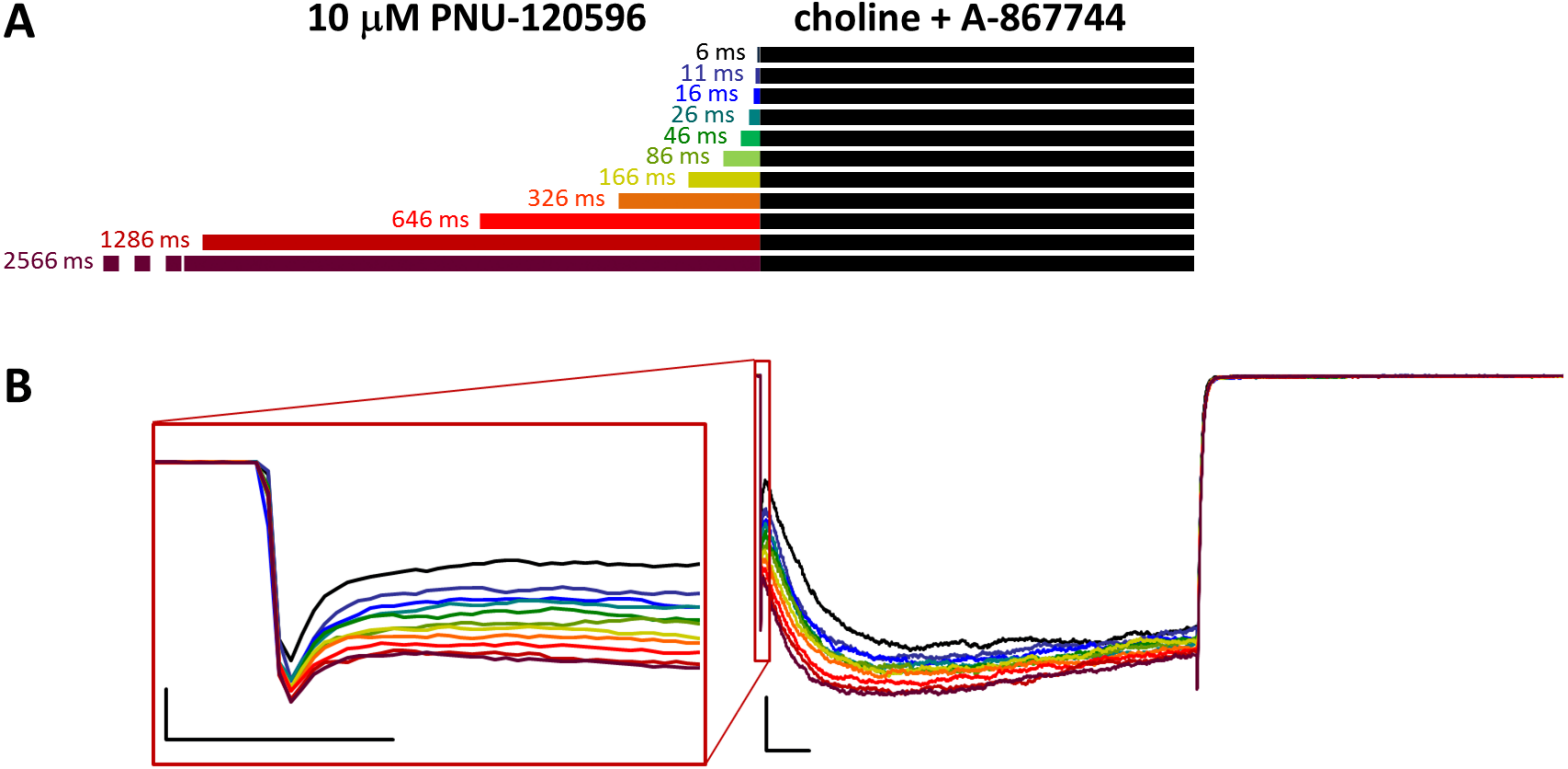
Effects of different lengths of 10 µM PNU-120596 preincubation on currents evoked by coapplied choline and 1 µM A-867744. (A) Perfusion protocol. (B) A typical example for the effect. Scale bars: 100 ms, 1 nA. Inset shows the first 20 ms of the agonist-evoked current at an expanded time scale. Scale bars: 10 ms, 1 nA.

During the experiments, the control extracellular solution was perfused continuously (flow rate of ∼1.66 ml/min). For fast drug application, we used piezoelectric-driven theta tubes (Burleigh LSS-3200 ultrafast solution switching system). The solution flow in the theta tubes was pressure controlled (DAD-12, ALA Instruments). Details of theta tube fabrication as well as the limitations of the solution exchange rates have been described previously (Pesti et al., 2014). The solution exchange rate also depends on the protocols used. In this study, the 10 to 90% solution exchange rates were between 1 and 3 ms. One side of the theta-tube contained the modulator, the other side contained either the agonist alone, or a mixture of agonist and modulator. In this study, we used three protocols to investigate the association, dissociation and displacement kinetics, as well as the modulated gating of the receptor. In the first protocol, we altered the length of preincubation by one of the modulators (*PRE* protocol); in the second, the length of coapplication (agonist and modulator together) was changed (*CO* protocol); and in the third, a fixed length of agonist + modulator coapplication was followed by different lengths of modulator perfusion (hence the name *POST* protocol), after which another agonist + modulator coapplication was executed to test the effect of modulator postapplication. The preincubation durations in the *PRE* protocol were 6, 11, 16, 26, 46, 86, 166, 326, 646, 1286, and 2,566 ms, followed by a 1,000 ms pulse of either agonist or agonist + modulator. Six ms was technically the possible shortest preincubation in this experimental protocol. Longer preincubation durations (up to 40 s) were tested in pilot experiments, but no significant change was observed after ∼2 s. This protocol was used in the experiments shown in Figs. 1 to 7. The effect of modulator preincubation reveals if the modulator is able to bind to the resting conformation, and if it does bind, it gives information on the rate of association.

**Figure 8:**
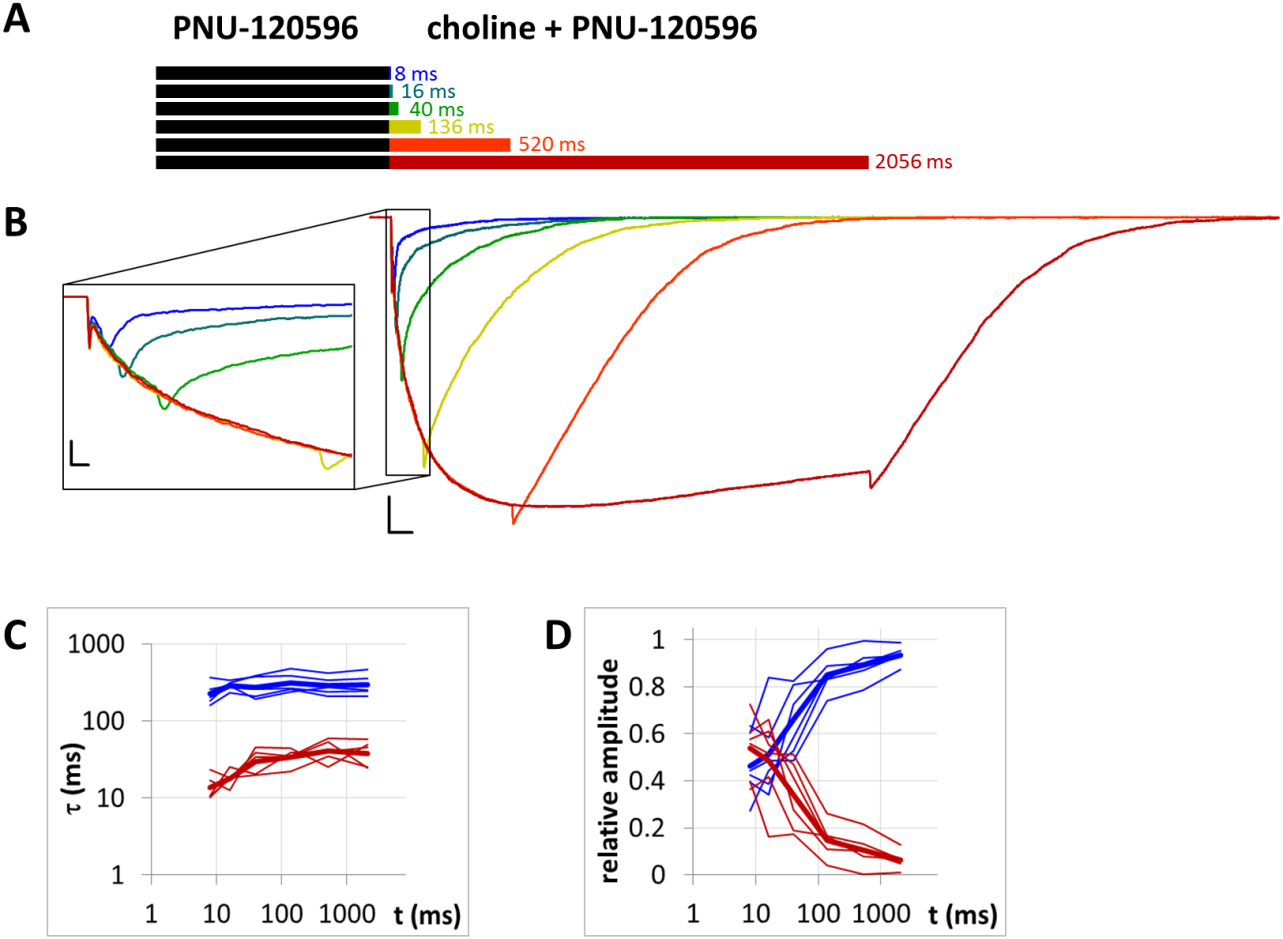
Currents evoked by different lengths of agonist and PNU-120596 coapplication after 1 s preincubation by PNU-120596. (A) Bars illustrate the perfusion protocol, 1 s of preincubation followed by different durations of agonist + modulator as indicated. Colors in (B) indicate corresponding lengths of coapplication as shown in (A). (B) An example for currents evoked by coapplied choline and PNU-120596 after 1 s of PNU-120596 preincubation. Scale bars: 100 ms, 1 nA. Inset shows the first 25 ms on an expanded time scale. Scale bars: 10 ms, 1 nA. Fast (red) and slow (blue) time constants of deactivation (C), and their respective contribution to the amplitude (D) after different lengths of choline and PNU-120596 coapplication. Thin lines show data from six individual measurements, thick lines show geometric mean for time constants, and arithmetic mean for relative amplitudes.

**Figure 9:**
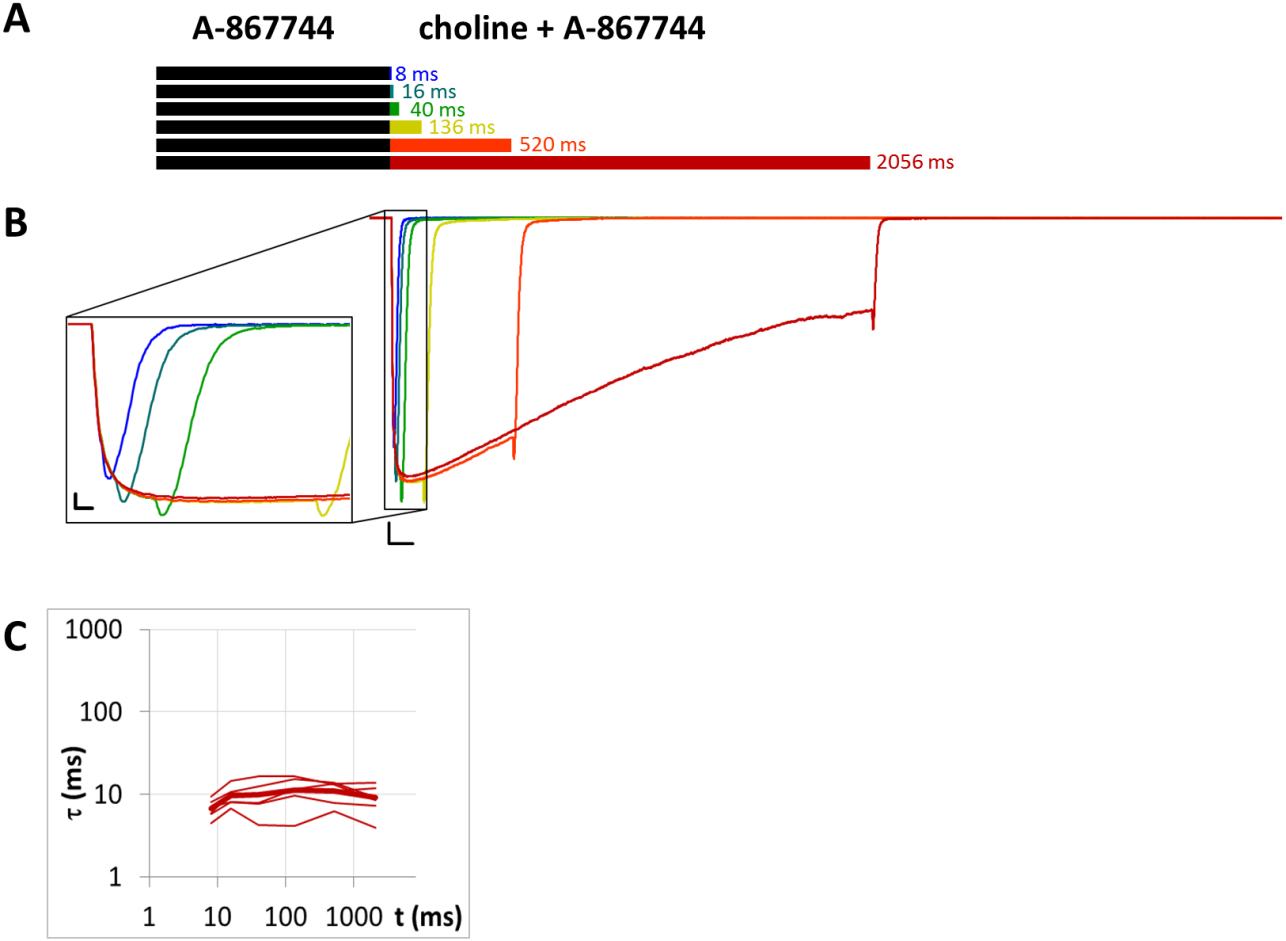
Currents evoked by different lengths of choline and A-867744 coapplication after 1 s preincubation by A-867744. (A) Perfusion protocol. (B) An example for the currents. Scale bars: 100 ms, 1 nA. Inset shows the first 25 ms on an expanded time scale. Scale bars: 10 ms, 1 nA. (C) The time constant of deactivation after different lengths of choline and A-867744 coapplication. Thin lines show data from six individual measurements, thick line shows geometric mean.

**Figure 10:**
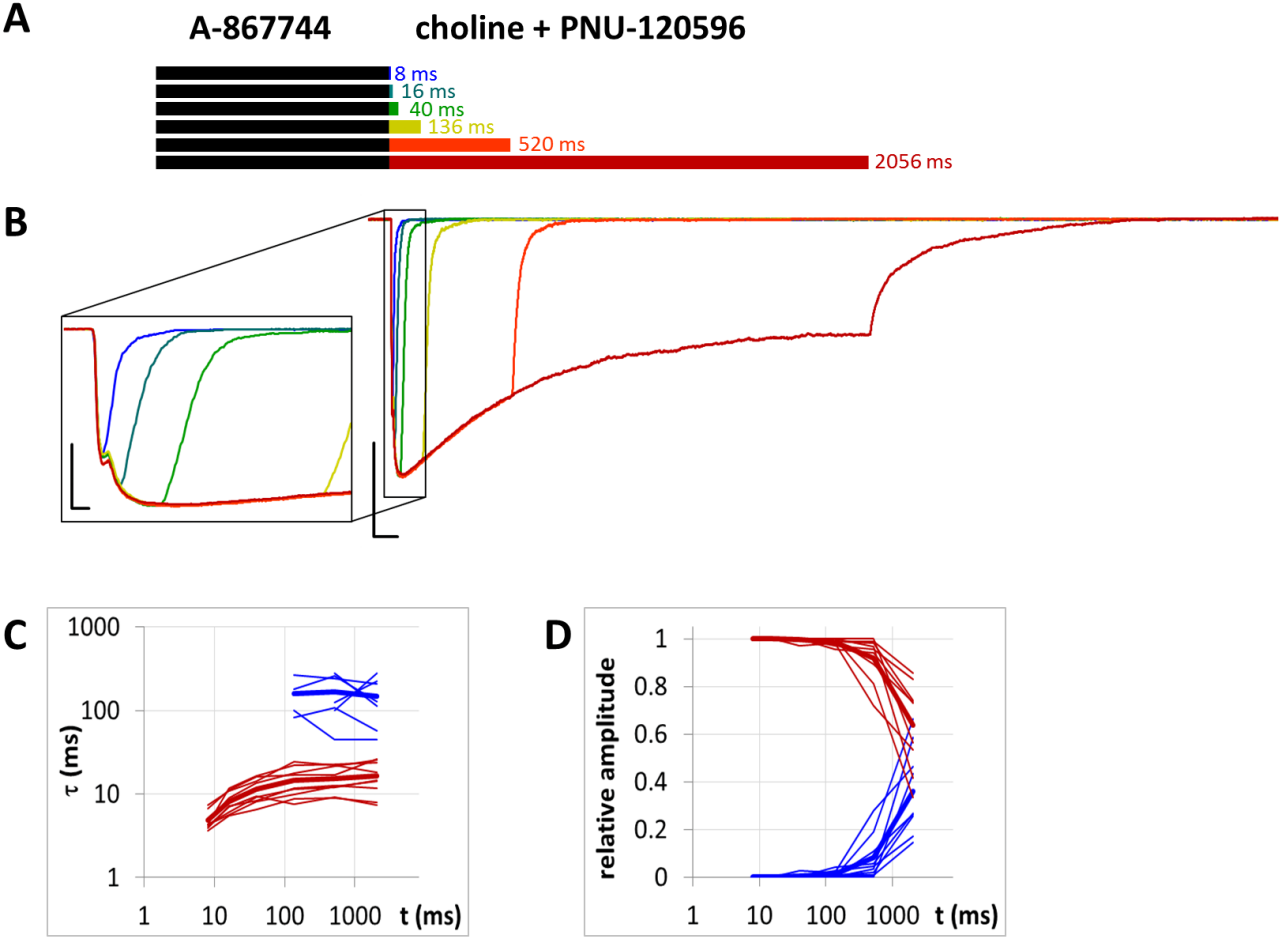
Currents evoked by different lengths of choline and PNU-120596 coapplication after 1 s preincubation by A-867744. (A) Perfusion protocol. (B) An example for the currents. Scale bars: 100 ms, 1 nA. Inset shows the first 25 ms on an expanded time scale. Scale bars: 10 ms, 1 nA. (C) and (D) Fast (red) and slow (blue) time constants of deactivation (C), and their respective contribution to the amplitude (D), after different lengths of choline and PNU-120596 coapplication. Thin lines show data from eight individual measurements, thick lines show geometric mean for time constants, and arithmetic mean for relative amplitudes.

**Figure 11:**
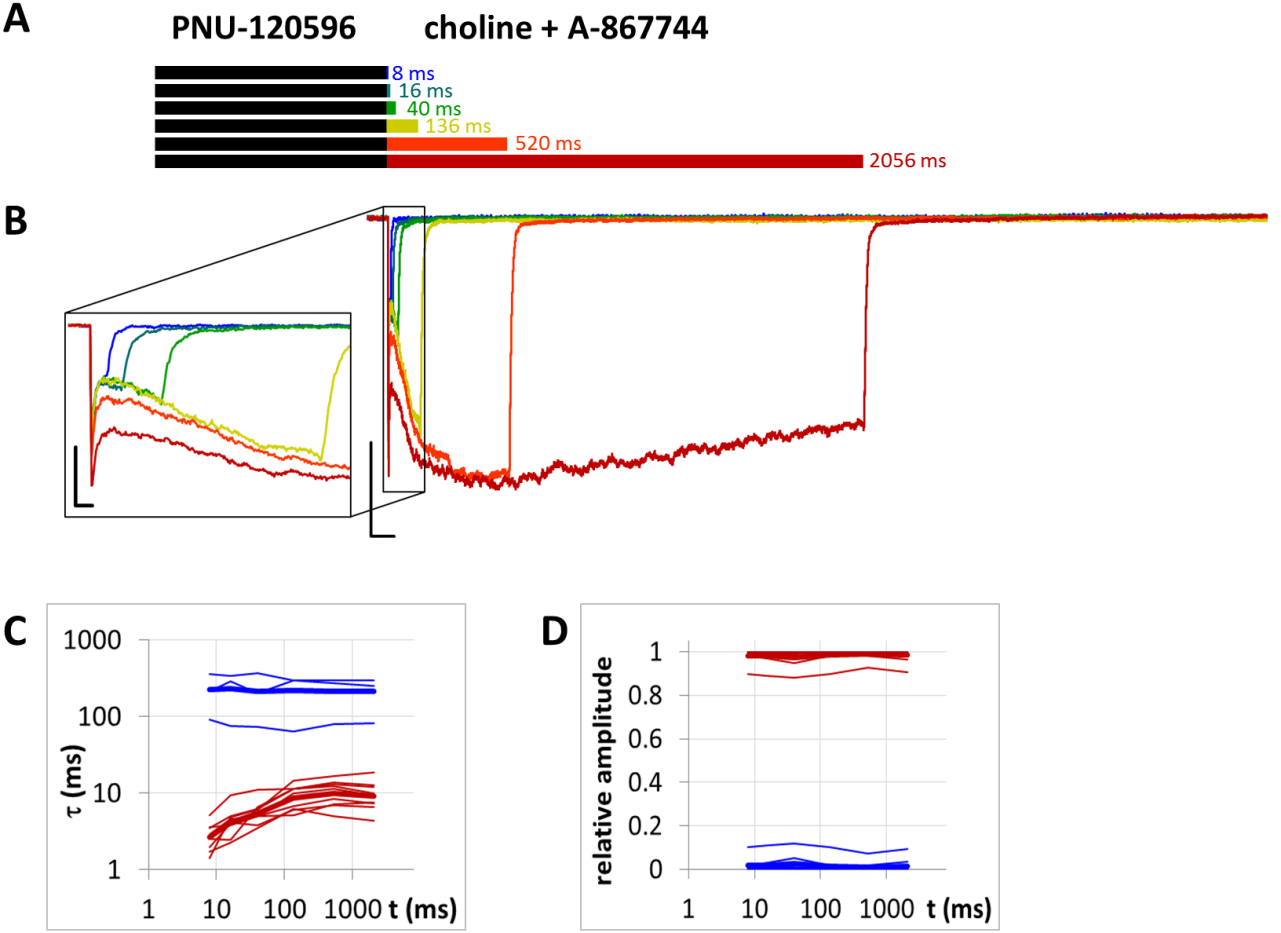
Currents evoked by different lengths of choline and A-867744 coapplication after 1 s preincubation by 1 µM PNU-120596. (A) Perfusion protocol. (B) An example for the currents. Scale bars: 100 ms, 1 nA. Inset shows the first 25 ms on an expanded time scale. Scale bars: 10 ms, 1 nA. (C) and (D) The time constant of deactivation (C), and their respective contribution to the amplitude (D), after different lengths of choline and A-867744 coapplication. Thin lines show data from six individual measurements, thick lines show geometric mean for time constants, and arithmetic mean for relative amplitudes.

In the *CO* protocol, a constant duration (1 s) of preincubation by one of the modulators was followed by coapplication for different durations (8, 16, 40, 136, 520, and 2,056 ms). We preincubated the cell with one of the modulators and then coapplied either the same or the other modulator with choline. This protocol was used in the experiments shown in Figs. 8 to 11.

**Figure 12:**
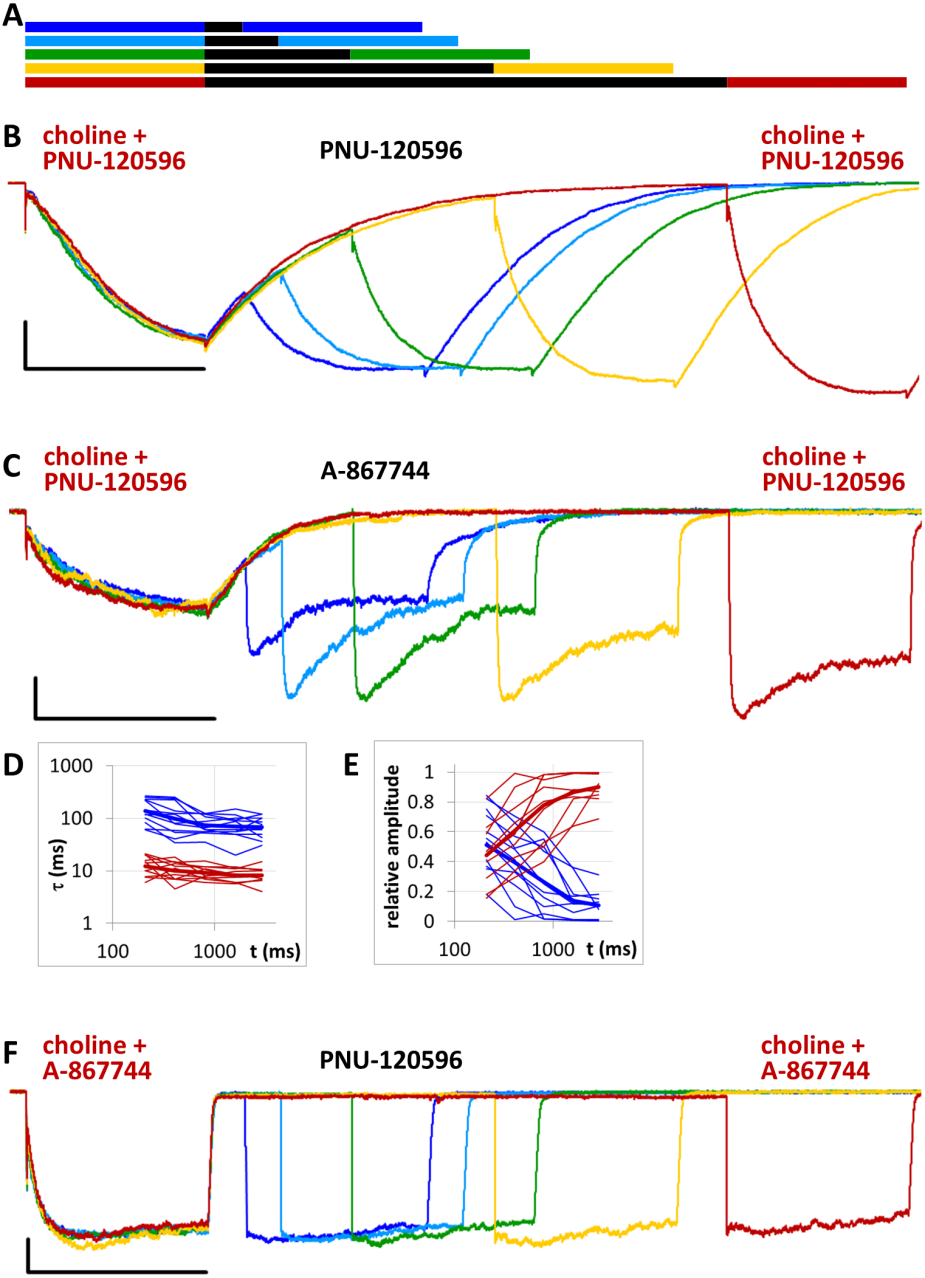
Effect of different durations of modulator application between two identical coapplications of modulator + agonist. (A) Schematic illustration of the perfusion protocol. (B) An example for currents evoked by coapplied choline and PNU-120596, intermitted by different durations of PNU-120596 application. Scale bars: 1 s, 1 nA. (C) An example for currents evoked by coapplied choline and PNU-120596, intermitted by different durations of A-867744 application. Scale bars: 1 s, 100 pA. (D) Time constants of deactivation after the second coapplication plotted against modulator pulse duration. Slow (blue) and fast (red) time constants are shown for *n* = 11 cells (thin lines), with their geometric mean (thick line). (E) Relative contribution of slow (blue) and fast (red) time constants as a function of modulator pulse duration. Thick lines show arithmetic mean. (F) An example for currents evoked by coapplied choline and A-867744, intermitted by different durations of PNU-120596 application. Scale bars: 1 s, 100 pA.

We used the *POST* protocol to test the displacement of one modulator by the other. The protocol consisted of two 1 s coapplication pulses, with different lengths of modulator pulses between them: 210, 410, 810, 1610, and 2,910 ms. The effect of the modulator was reflected by the difference between the two coapplication-evoked currents. This protocol is illustrated in Fig. 12A.

### Analysis

Curve fitting was performed with the Solver function of Microsoft Excel. Decay phases were fit with a standard monoexponentia function:

\begin{eqnarray*}I(t)=({I}_{max}-{I}_{min})~\ast ~\exp \nolimits (-t/\tau )+{I}_{min}, \end{eqnarray*}

or double-exponential functions:

\begin{eqnarray*}I(t)=({I}_{max}-{I}_{min})~\ast ~[{A}_{\mathit{1}}~\ast ~\exp \nolimits (-t/{\tau }_{1})+{A}_{\mathit{2}}~\ast ~\exp \nolimits (-t/{\tau }_{2})]+{I}_{min} \end{eqnarray*}

where *τ*_1_ and *τ*_2_ are the time constants and *A*_1_ and *A*_2_ are their respective contributions to the amplitude. To measure the maximum slope values at the initial fast component of the currents, the slope of each current was plotted, using an average over a 200 µs time window, in order to minimize the effect of noise.

### Simulations

Kinetic simulations were based on a set of differential equations with the occupancy of each receptor state (i.e., the fraction of the receptor population in that specific state) given by the following equation:

\begin{eqnarray*} \frac{d{S}_{i}(t)}{dt} =\sum _{j}^{n}{S}_{j}(t)^{\ast }{k}_{ji}-{S}_{i}(t)^{\ast }{k}_{ij} \end{eqnarray*}

where *S*_*i*_(*t*) is the occupancy of a specific state at time t, *S*_*j*_(*t*) is the occupancy of a neighboring state, *n* is the number of neighboring states, and *k*_*ij*_ and *k*_*ji*_ are the rate constants of the transitions between the neighboring states. Simulations were performed using Berkeley Madonna v8.0.1 (http://www.berkeleymadonna.com).

## Results

### Preincubation with A-867744 was effective on resting receptors

The *PRE* protocol was used to study the effect of perfusing A-867744 before choline application (Fig. 1B). Preincubation was evidently effective right from the initial phase of the current. Choline alone evoked a rapidly desensitizing peak of current. When choline was coapplied with 1 µM A-867744, this peak was unchanged, and the effect of modulator was only seen in the prolonged activation after the peak. The shortest (6 ms) preincubation did not affect the onset slope or the amplitude of the peak (slope: 95.2 ± 3.5% of control, amplitude: 101.1 ± 3.6% of control; *n* = 9), but with longer (>30 ms) preincubation durations, the current gradually transformed. At low temporal resolution, an increased amplitude was apparent (248 ± 31%; *n* = 8, *p* = 0.009), as shown in Fig. 1B. However, at high temporal resolution, it was evident that the initial phase was not enlarged but instead transformed (Fig. 1B inset). The rate of activation decreased, indicating that some of the current that was carried by fast-opening unmodulated receptors was replaced by current carried by the modulated receptors opening at a lower rate. Maximum slope values ranged between 950 and 6,352 pA/ms in the six ms preincubation traces. (The large variance was due to the variance in peak amplitudes; expressed relative to the peak amplitude, the maximum slope values were 1.79 ± 0.28 ms^−1^). For the 2,566 ms preincubation, the maximum slope values decreased to 33.5 ± 4.8% of the 6 ms value (*n* = 7, *p* = 0.007). For the sake of clarity, we only show the slope values for the shortest and longest preincubation (Fig. 1C), but the slope values always changed monotonically. Each line shows a pair of slope values measured in the same experiment. This is significant because it indicates that—unlike in the case of PNU-120596 as shown below—the modulator is able to bind to the receptor in the resting state.

The maximum amplitude of the modulated current was reached within ∼50 ms, after which the currents decayed monoexponentially with the time constant of 233 ± 33.1 ms at 2,566 ms preincubation; for the other preincubation durations see Fig. S1E; and after longer preincubations, the current did not reach zero within the 1 s of agonist application in 4 out of the 7 cells. Upon termination of agonist application, the current rapidly decayed to baseline with a deactivation time constant of 4.27 ± 0.33 ms. The deactivation time constant was not significantly affected by the preincubation duration (Fig. S1D).

The effectiveness of modulation is reflected by changes in the amplitude and the net charge flux value (area under curve, AUC). Amplitude values are expressed as relative to the amplitude of the peak component. AUC values are the charge carried throughout the 1 s of coapplication, divided by the charge carried during the peak component. For the cases in which the peak component did not decay to the baseline, we calculated the charge carried during the first 3 ms. The effects of preincubation are summarized in Table 1. Data from the individual experiments for all preincubation times are plotted in Figs. S1B and S1C.

When A-867744 preincubation was followed by the coapplication of A-867744 and choline (Fig. 2A), both the increased amplitude (263 ± 41%; *n* = 8, *p* = 0.002) and the deceleration of onset (maximum slope values decreased to 53.6 ± 8.7%; *n* = 8, *p* = 0.025; Fig. 2B inset and Fig. 2C) were similar. Currents reached their maxima within ∼50 ms and decayed exponentially afterwards, but in this case, the decay was slower (the time constant was 361 ± 23.7 ms; Fig. S2E), and a significant fraction of the receptors were still open at the end of the 1 s coapplication. This also meant that net charge flux values (AUC) were higher (see Table 1 and Fig. S2C). Deactivation upon termination of agonist and modulator perfusion was fast, with a time constant of 6.16 ± 1.54 ms, which did not depend on the preincubation duration (Fig. S2D).

For the amplitude and AUC data, see Table 1 and Figs. S2B and S2C.

### Preincubation with PNU-120596 was ineffective on resting receptors

When PNU-120596 was preapplied before 10 mM choline application (Fig. 3), both the amplitude and the onset slope of the initial peak component (evoked by the agonist alone) were unchanged.

This shows that during the ∼2.5 s preincubation period, PNU-120596 was unable to exert its modulatory effect on the resting receptors (Szabo et al., 2014). PNU-120596-modulated receptors have radically longer open and closed times (Williams, Wang & Papke, 2011a; DaCosta et al., 2011) and are, therefore, unable to produce a fast peak component. Current during the initial peak component had to be conducted by unmodulated receptors. However, immediately after the initial component, a definite positive modulation occurred, especially after longer preincubation times. There was prolonged activation during the agonist pulse, which came from the characteristic effect of PNU-120596: a radical prolongation of opening, which comes in long bursts (mean burst times ≈1–2 s). This was evident in the recordings from outside-out patches with relatively few receptors, where long bursts of single-channel openings during post-modulator agonist application were detectable (as shown in Szabo et al., 2014). The relative amplitude of the prolonged component never exceeded the peak component; it reached only 48.9 ± 5.8% (*n* = 9) of the peak amplitude at the longest preincubation time (Fig. S3C), after which it decayed exponentially with a time constant of 365 ± 88.8 ms (Fig. S3E) and reached baseline before the 1 s choline pulse was terminated in 5 out of 9 cells/patches. The net charge carried by this late component increased to only 112 ± 17.2 times the net charge carried by the peak component (Table 1 and Fig. S3D). In summary, the initial peak component was unchanged, indicating that the modulator binding site was not occupied at the beginning of the agonist pulse. Afterwards, some prolonged activation occurred, indicating that although the modulator molecules were washed out from the extracellular aqueous phase, they must have accumulated within the membrane phase, from where they could access the allosteric binding site when the desensitized conformation allowed it. Alternatively, PNU-120596 molecules might have accessed their binding pocket in the resting state but could not assume the correct orientation needed for modulatory action until the receptors gated into desensitized conformation.

When PNU-120596 preincubation was followed by the coapplication of PNU-120596 and choline, the evoked currents were radically different: the modulator caused a large potentiation (amplitude increased to 410 ± 27%, net charge increased 2183-fold, *n* = 6), while preincubation had only a minor effect (Fig. 4, Figs. S4C and S4D, Table 1).

The only evident effect of preincubation was the acceleration of the second, slower current component (from 272 ± 52.2 to 130 ± 18.4 ms, Fig. S4F), as we described earlier with 10 µM PNU-120596 (Fig. 3 in Szabo et al., 2014). Deactivation after agonist and modulator coapplication was slow (279 ± 22 ms, Fig. S4E), and it did not change significantly by preincubation.

### Preincubation with 1 μM A-867744 prevented the subsequent effects of coapplied 1 μM PNU-120596

We intended to study the interaction of the two modulators; therefore, we tested whether preincubation with one modulator could alter the currents evoked by coapplication of the other modulator with choline. There are three key features by which one can judge which modulator’s influence is present (compare Figs. 2 and 4). First, the initial phase of the current was unchanged by PNU-120596, while A-867744 enlarged and broadened it, causing the separate peak component to “dissolve” into the enlarged current. Second, in the presence of PNU-120596, the onset of modulation was slow, current amplitude continued to increase throughout the first 500 ms. In contrast, in the presence of A-867744, the maximum amplitude was reached within the first 50 ms, after which the current decayed exponentially. Third, deactivation was slow after choline and PNU-120596 coapplication (time constant ≈ 300 ms) but fast (time constant <10 ms) after choline and A-867744 coapplication. From these three features, one can judge which of the two modulators exerts the dominant effect at the beginning of the agonist pulse, throughout the agonist application, and at its termination.

When we preincubated cells with A-867744 before the coapplication of choline and PNU-120596 (Fig. 5), the influence of PNU-120596 was gradually overcome by the influence of A-867744, as shown both by the decay of the current during the coapplication (agonist + modulator) and the rapid decay after the coapplication.

After longer preincubation times with A-867744, PNU-120596 could not exert its characteristic effect despite being coapplied with the agonist for 1 s. As the preincubation duration increased, the pattern of evoked currents changed in all three features. First, the initial peak current was transformed by the effect of the preapplied A-867744 as we had seen above. The current onset became slower (Fig. 5C) (by the 2,566 ms preincubation, the slope decreased to 40.8 ± 4.3% of the six ms value, *n* = 7, *p* = 0.002), but the amplitude of the current increased (268 ± 30%, *n* = 7, *p* = 0.004, Fig. S5B). Second, during the 1 s agonist perfusion, the slow increase due to PNU-120596 coapplication was overcome by the decay typical of A-867744. Third, the deactivation after agonist and modulator coapplication was accelerated; it showed both a fast and slow component throughout the experiment (13.6 ± 0.95 and 181 ± 8.7 ms, respectively, Fig. 5D), but the contribution of the fast component gradually became predominant (from 5.9 ± 1.5% to 84.3 ± 3.8%, Fig. 5E). Exponential fits to the relative amplitude curves (Fig. 5E) show that development of the fast component of deactivation occurred with a time constant of 246 ± 72 ms (*n* = 7), which is similar to that of the amplitude potentiation (Table 1, Fig. S5B). The occurrence of a fast component of deactivation indicates that the preapplied A-867744 was not removed from its binding site within the 1 s perfusion of PNU-120596 and choline; therefore, PNU-120596 was hindered either in binding or exerting its modulatory effect (see next section).

In summary, the effects of even a few tens of milliseconds of A-867744 preincubation were already visible, mostly by its interference with PNU-120596-mediated modulation, as the characteristic pattern of PNU-120596-modulated current could not develop. In spite of this, PNU-120596 could associate to its binding site, as evidenced by the slow component of deactivation after coapplication. At preincubation durations longer than ∼200 ms, the influence of A-867744 became dominant, which was seen in the enlarged initial component, the decaying shape of the choline and PNU-120596 coapplication-evoked current, and the predominantly fast deactivation after the agonist-modulator pulse.

### Competition for the same binding site, or different efficacies of modulation?

Does the interference of the two modulators necessarily mean that they must share the same binding site? It is also possible that they have separate binding sites but interact on the level of modulating channel gating. Which mechanism is more likely? If we suppose that the two modulators compete for the same binding site, then it is natural that PNU-120596 can fully associate during the 1 s period when the binding sites are practically unoccupied by A-867744 (see the black and blue traces in Fig. 5B), and therefore PNU-120596-evoked modulation can dominate the deactivation phase. On the other hand, when the binding sites have already been occupied by the preapplied A-867744, the association of PNU-120596 is hindered. If, however, we suppose that there are distinct binding sites, then we must also suppose that A-867744 is much more effective in determining decay kinetics, because even after 1 s of partial dissociation, A-867744 can still overcome the effects of PNU-120596. If the latter is the case, then A-867744 should overcome even higher concentrations of PNU-120596 because the binding of more PNU-120596 molecules would not affect the presence of A-867744 molecules at their own binding site. We tested this hypothesis by increasing the PNU-120596 concentration to 10 µM.

**Table 1:** Summary of main findings. The table summarizes the most important findings of this study. Rows 1 to 7 show results obtained with the “PRE” protocol, as shown in Figs. 1 to 7. Rows 8 to 11 show results of the “CO” protocol, as shown in Figs. 8 to 11. Rows 12 to 14 show results of the “POST” protocol, as shown in Fig. 12. Concentration of modulators is 1 µM unless shown differently. Concentration of choline was 10 mM. Columns 1 to 3 describe the perfusion used in that specific experiment. Red “ Δt” signs indicate the part of the protocol that was changed in duration. Effects of these changes are shown in columns 4 to 6. Bold fonts indicate significant changes (*p* < 0.01).

***Pre-applied***	***Co-applied***	***Post-applied***	**amplitude potentiation**	**AUC potentiation**	**change in***τ*_deact_
A-867744 Δt	choline		**248** ± 31% *τ* = **332** ± 43 ms	**311** ± 28-fold *τ* = **303** ± 50 ms	92 ± 19%
A-867744 Δt	choline A-867744		**263** ± 41% *τ* = **169** ± 76 ms	**633** ± 96-fold	105 ± 14%
PNU-120596 Δt	choline		99 ± 7%	**112** ± 17-fold *τ* = **2 813** ± 902 ms	–
PNU-120596 Δt	choline PNU-120596		96 ± 2% (peak) **410** ± 27% (slow component)	**2,183** ± 110-fold	104 ± 2%
A-867744 Δt	choline PNU-120596		**268** ± 33% *τ* = **182** ± 99 ms	**928** ± 91-fold	fast decay overcomes slow decay *τ* = **246** ± 72 ms
A-867744	choline 10 µM PNU-120596 Δt		**255** ± 52%	**1,820** ± 1,059-fold	fast decay starts to manifest with *τ* >**5,865** ± 2,398 ms
10 µM PNU-120596	choline A-867744 Δt		129 ± 18%	**1,076** ± 438-fold	slow component absent or minimal
PNU-120596	choline PNU-120596 Δt		95 ± 8% (peak) **306** ± 93% (slow component)	**1,554** ± 443-fold	association during co-application *τ* = **97.8** ± 60.2 ms
A-867744	choline A-867744 Δt		–	–	135 ± 20%
A-867744	choline PNU-120596 Δt		–	–	slow component appears with: *τ* =**5,304** ± 1,325 ms
PNU-120596	choline A-867744 Δt		108 ± 7% (peak) 122 ± 10% (slow component)	**768** ± 80-fold	association during co-application *τ* = **47.8** ± 6.65 ms
	choline PNU-120596	PNU-120596 Δt	106 ± 21% (peak) **260**± 45% (slow component) **293** ± 74% (2nd pulse slow c.)	**1,195** ± 206-fold (1st pulse) **1,366** ± 230-fold (2nd pulse)	94 ± 11%
	choline PNU-120596	A-867744 Δt	113 ± 13% (peak) **190**± 34% (slow component) **481** ± 102% (2nd pulse, 2,910 ms)	**647** ± 76-fold (1st pulse) **1,704** ± 266-fold (2nd pulse, 2,910 ms)	fast decay overcomes slow decay *τ* = **456** ± 155 ms
	choline A-867744	PNU-120596 Δt	78 ± 6% (peak) 141 ± 20% (slow component) 147 ± 16% (2nd pulse, 2910 ms)	**770** ± 147-fold (1st pulse) **922** ± 162-fold (2nd pulse, 2910 ms)	99 ± 1%

### Preincubation with 1 μM A-867744 did not prevent the effects of coapplied 10 μM PNU-120596

We found that increasing the PNU-120596 concentration to 10 µM effectively diminished the fast component of deactivation. This fast component was either completely missing (in three of the seven cells) or, in the remaining four cells, its appearance required at least 300 ms of preincubation, and its contribution to the amplitude was only 12.0 ± 3.1% (Fig. 6D). When single exponentials were used to fit the relative amplitude curves (Fig. 6E), the time constant was 5,865 ± 2,398 ms (*n* = 4), which is an underestimation because we could not include cells where the fast component did not appear. The effect of A-867744 preincubation was evident at the initial phase of the current (Fig. 6B), which had an increased amplitude (255 ± 52%, *n* = 7, *p* = 0.04) and a decreased slope (32.4 ± 7.0%, *n* = 7, *p* = 0.007); however, the shape of the current during the 1 s of coapplication was neither monotonously increasing (a sign of modulation by PNU-120596) nor exponentially decreasing after the initial ∼100 ms (a sign of modulation by A-867744), but instead, PNU-120596-like characteristics were gradually overcome by A-867744-like characteristics. Neither the relative net charge nor the maximum amplitude of the initial phase increased monotonously (Fig. S6B and S6C), indicating interference between the two modulators. These results suggest that the two modulators compete for the same binding site and can displace each other.

### Preincubated PNU-120596 is rapidly displaced by A-867744

In the reverse experiment, when cells were preincubated in PNU-120596 before the coapplication of choline and A-867744, the preincubation did not radically change the shape of the coapplication-evoked currents. Because PNU-120596 was mostly ineffective during preincubation and because of the apparent difference in affinities (or possibly in stoichiometry, see below), we used 10 µM PNU-120596 for preincubation. The initial peak component did not change significantly by the increased duration of preincubation (Figs. 7B and S7B).

However, the effects of preincubation could be clearly observed on the shape of the currents. Choline-A-867744 coapplication-evoked currents showed mixed features: a slow onset in the first ∼2–300 ms, which is characteristic of PNU-120596-mediated modulation, and this onset was slightly accelerated with longer PNU-120596 preincubation times. After ∼2–300 ms, however, the current started to decay with a time constant of 889 ± 227 ms, indicating the influence of A-867744, although the typical rate of decay during A-867744-mediated modulation was not reached (∼200 to 400 ms). We can conclude that throughout the 1 s of coapplication, both modulators exerted their effect, which suggests a mixed occupancy of the binding sites. Remarkably, however, deactivation at the end of the 1 s pulse of A-867744 and choline was fast and monoexponential (time constant was 9.56 ± 0.29 ms, *n* = 6), as if PNU-120596 had been completely displaced by A-867744. What can be the reason for the full 1 s current showing mixed characteristics up to the very end, while the offset showed the influence of A-867744 only? We suppose that the explanation may be different stoichiometries for the two modulators. If occupancy of four or five binding sites per receptor by PNU-120596 is a prerequisite for slow deactivation, then it is possible that 1 s of A-867744 perfusion was enough to displace at least two PNU-120596 molecules from almost all of the receptors. Therefore, it seemed necessary to investigate the dynamics of the modulators competing for the binding site.

### The dynamics of modulator displacement

To study the dynamics of competitive displacement between the two modulators, we used the *CO* protocol: constant duration (1 s) of preapplication preceded different durations (8, 16, 40, 136, 520, and 2,056 ms) of coapplication. We preincubated the cells with one of the modulators and then coapplied either the same or the other modulator with choline.

### The dynamics of modulator binding in the case of PNU-120596

When the same modulator, PNU-120596 (at a 1 µM concentration), was preincubated and then coapplied with choline (Fig. 8B), we expected that the currents would reflect the following four specific characteristics of the PNU-120596 effect (Szabo et al., 2014): (i) At the start of coapplication, there would be a peak component (<5 ms in width) that is identical to the current evoked by the agonist alone. Preincubation with PNU-120596 should not change this component; (ii) PNU-120596-induced modulation would develop slowly, with time constants of ∼100 to 300 ms, reaching a maximum within ∼500 ms; (iii) At the end of the coapplication, there would be a transient (∼10 ms in width) increase in the current (“rebound component”, see Szabo et al., 2014) caused by relief from the agonist block; and (iv) Deactivation would be slow and monoexponential, with time constants of ∼200 to 400 ms. The first three characteristics were indeed observed. The fourth (slow deactivation) was not observed after the short coapplication durations; the decay was faster and biexponential. At the shortest coapplication duration (8 ms), the slow component contributed 46 ± 5.5% of the amplitude and with longer coapplication durations, the contribution of the slow time constant continued to increase, up to 93.6 ± 1.5% (after 2,056 ms). In addition to the increasing contribution of the slow component (Fig. 8D), the decay slowed by the expansion of the fast component from 13.6 ± 2.4 ms to 38.2 ± 6.2 ms (Fig. 8C) (a 281% change, *p* = 0.033, *n* = 6), while the time constant of the slow component did not change significantly. The existence of the fast component at short coapplication durations indicates that it took a considerable amount of time to reach the occupancy level that allows for the manifestation of the modulation in the entire receptor population, even though preincubation was sufficiently long (1 s). This indicates that the binding of PNU-120596 to the receptor in resting conformation was indeed hindered, and the development of positive modulation required an encounter with the receptor in desensitized conformation for ∼20 to 200 ms. The development of the slow decay component was fit to an exponential function in each cell, and the time constant was 97.8 ± 60.2 ms (*n* = 6). The onset of the PNU-120596-mediated component follows practically the same time course. As shown in Fig. 8B, the development of the slow delay and the onset of the modulated current proceeded in parallel. The relative amplitude of the slow component (as compared to the amplitude of the peak component) and the charge conducted by it (as compared to the charge conducted during the peak component) were similar to the values found in the *PRE* experiment (Table 1).

### Unhindered association of A-867744 to resting state receptors

When 1 µM A-867744 was both pre- and coapplied (Fig. 9), the evoked current bore the characteristics of A-867744-evoked modulation, as described above. The time constant of deactivation was 6.83 ±0.71 ms at the shortest and 9.12 ± 1.40 ms at the longest coapplication duration, which were not significantly different (*p* = 0.14, *n* = 6). The decay after the short coapplication durations was, however, slower than the deactivation upon termination of agonist application, indicating that A-867744 could bind to resting receptors and exert its effect from the very beginning of coapplication. This was also shown by the fact that—as we have discussed above—the distinct peak of unmodulated choline-evoked current did not occur; instead, we only saw the characteristic A-867744-modulated current, with a slightly slower onset and augmented amplitude. For this reason, the relative amplitude and relative AUC values were not calculated for Table 1.

### Slow displacement of A-867744 by PNU-120596

When A-867744 (1 µM) was preapplied before choline and PNU-120596 (1 µM) coapplication, the effect of A-867744 dominated the pattern of evoked currents. This was evident already at the initial phase of the currents: the isolated peak component was absent, replaced by an augmented, but somewhat slower, A-86774-modulated onset (Fig. 10). The shape of the current also reflected the effect of A-867744 rather than that of PNU-120596: the maximum was reached within 40–80 ms, which was followed by exponential decay with a time constant of 400–800 ms. In addition, the deactivation after coapplication at the intermediate coapplication intervals (16 and 40 ms, as well as 136 ms in five out of nine cells) also solely reflected the effect of A-867744: the current decay was mono-exponential, with a time constant of 10.9 ± 1.02 ms. (Interestingly, at the shortest interval, the time constant was 4.83 ± 0.44 ms. This might reflect a situation where neither of the modulators could effectively exert their effect.) Only after coapplication with PNU-120596 for as long as 520 or 2,056 ms did the slow component of the post-coapplication decay appear (Figs. 10B–10D). Its contribution reached 36.2 ± 6.1% after 2,056 ms of coapplication. The exponential fit of the development of the slow component (Fig. 10D) gave a time constant of 5,304 ± 1,325 ms (*n* = 9), which is >50 times slower than that which was observed after PNU-120596 preincubation (Fig. 8D). Therefore, it seems obvious that the presence of A-867744 at its binding site effectively hindered PNU-120596 binding. There is an apparent contradiction between the fast deactivation of the A-867744-modulated current (with a time constant of ∼10 ms), and the modulation that persists up to ∼1,000 ms after completion of A-867744 perfusion. This indicates that deactivation is evoked by the dissociation of the agonist and not the modulator. Despite the fast deactivation, A-867744 molecules typically remain at the binding site for more than a second. As mentioned above, one reason for the slow displacement of A-867744 molecules by PNU-120596 molecules may be the different stoichiometry of the modulators. Mixed occupancy receptors may manifest A-867744-like properties, and the characteristics of PNU-120596 may only appear when all five bound A-867744 molecules are replaced by PNU-120596 molecules.

### Unhindered association of A-867744 to receptors preincubated with PNU-120596

We tested this explanation by the reverse arrangement. When PNU-120596 was preapplied before A-867744 and choline coapplication (Fig. 11), we expected that in the resting state, modulator binding sites would not be able to reach sufficient occupancy by PNU-120596, and therefore, we would not see any obvious sign of PNU-120596-mediated modulation. As expected, the overall shape of the currents was similar to the effect of coapplied A-867744 and choline with minimal A-867744 preapplication (Fig. 2B, black trace). The initial peak component was preserved (Table 1), after which the onset of the current was slow, reaching its maximum at 331 ± 38 ms (*n* = 9), where the maximum amplitude was not significantly higher than the amplitude of the peak component (Table 1). Then, the current decayed at a slow rate (*τ* ≫ 1,000 ms). This should not be interpreted as a proof of PNU-120596 hindering A-867744 access to the binding site because A-867744 and choline without long preincubation evoked similar currents (Fig. 2B, black and blue traces). The fact that PNU-120596 was unable to bind to resting receptors is also shown by the fact that the deactivation was fast, even after the shortest (8 ms) coapplication (Figs. 11B and 11C), where hardly any A-867744 binding had occurred. In fact, deactivation after the shortest coapplication periods was especially fast (2.65 ± 0.38 ms at 8 ms, 4.15 ± 0.68 ms at 16 ms), faster (*p* < 0.001 in both cases) than in the case of the coapplication of A-867744 and choline after preincubation with A-867744 (6.83 ± 0.72 ms at eight ms, 9.78 ± 1.13 ms at 16 ms; Fig. 9C). This indicates that at these shortest coapplication durations, the unmodulated receptors (which deactivate faster) were predominant, and from the monoexponential fits to the fast time constant vs. coapplication duration plot (Fig. 11C), the association of A-867744 proceeded with a time constant of 47.8 ± 6.65 ms. Since we obtained similar association rates for the resting receptors, there is no reason to suppose that interference with the already bound PNU-120596 might have hindered the access of A-867744 to the binding site. A minor slow component of deactivation was detectable in four out of the nine cells, and on average, it contributed only 2.53 ± 0.6% to the amplitude. Note that without subsequent A-867744 application, PNU-120596 was able to evoke some modulation even when it was only preapplied (Fig. 3B); however, it seemed to lose its efficacy when pre-incubation was followed by A-867744 coapplied with choline. This confirms our hypothesis that resting receptors cannot host PNU-120596 molecules, and the effects observed from the preincubated PNU-120596 (see Fig. 3) are mostly due to the accumulation of the modulator in the membrane phase.

### Displacement of coapplied modulators

Since we have seen that association of PNU-120596 to resting receptors is hindered, the only way to examine the displacement of PNU-120596 by A-867744 is by perfusing A-867744 after the coapplication of choline and PNU-120596. Therefore, we used the *POST* protocol, which consisted of two 1 s coapplication pulses with different lengths of a modulator pulse between them: 210, 410, 810, 1610 and 2,910 ms (Fig. 12A). The case where PNU-120596 was perfused between the two choline + PNU-120596 coapplications is shown for comparison (Fig. 12B). Perfusion of PNU-120596 between the two coapplications accelerated the onset of the second pulse, as has been shown before (Szabo et al., 2014). This only caused a minor increase in the amplitude or net charge carried by the second pulse compared to the first one (Table 1).

When we perfused A-867744 between the two choline + PNU-120596 coapplications, the shape of the second coapplication-evoked current was radically altered (Fig. 12C). Even after the shortest 210 ms application of A-867744, the shape of the current reflected the characteristics of A-867744-mediated modulation: the initial peak component was lost and was replaced by a slightly slower onset with an augmented amplitude (287 ± 56%, *p* < 0.0001, *n* = 11), which continued to increase with longer A-867744 perfusion, reaching 481 ± 102% at 2,910 ms (Table 1). The effect of undissociated PNU-120596 molecules, however, was also evident, because a slow component of deactivation after the second coapplication-evoked current persisted, especially with shorter A-867744 applications (Figs. 12D and 12E). This effect was gradually overcome by the fast component as the duration of A-867744 application increased (the contribution of the fast component to the amplitude increased from 44 ± 6.5% at 210 ms to 90 ± 3.1% at 2,910 ms).

It is important to note that the presence of A-867744 was unable to change the course of decay after the first pulse of choline and PNU-120596. This, however, cannot indicate the inability of A-867744 to associate to its binding site, because at the same moment, A-867744 could change the onset phase of choline + PNU-120596 evoked currents and even the decay phase after 1 s of coapplication. It is evident that the modulators must exert different efficacies toward the distinct features of evoked currents, and therefore, the occupancy of the modulator binding sites cannot be judged by a single feature.

PNU-120596, after choline + A-867744 coapplication, was also unable to alter the time course of deactivation (Fig. 12F) and had no detectable effect on the second coapplication-evoked current. This does not mean that the second current was identical to the first one. The initial phase was changed by the first A-867744 application, indicating that even the 2,910 ms perfusion of PNU-120596 was not enough to completely displace A-867744 at the binding site. However, a limited association must have occurred, as shown by the pattern of the current evoked by the second pulse, which showed mixed characteristics.

The main findings of the experiments are summarized in Table 1.

**Figure 13:**
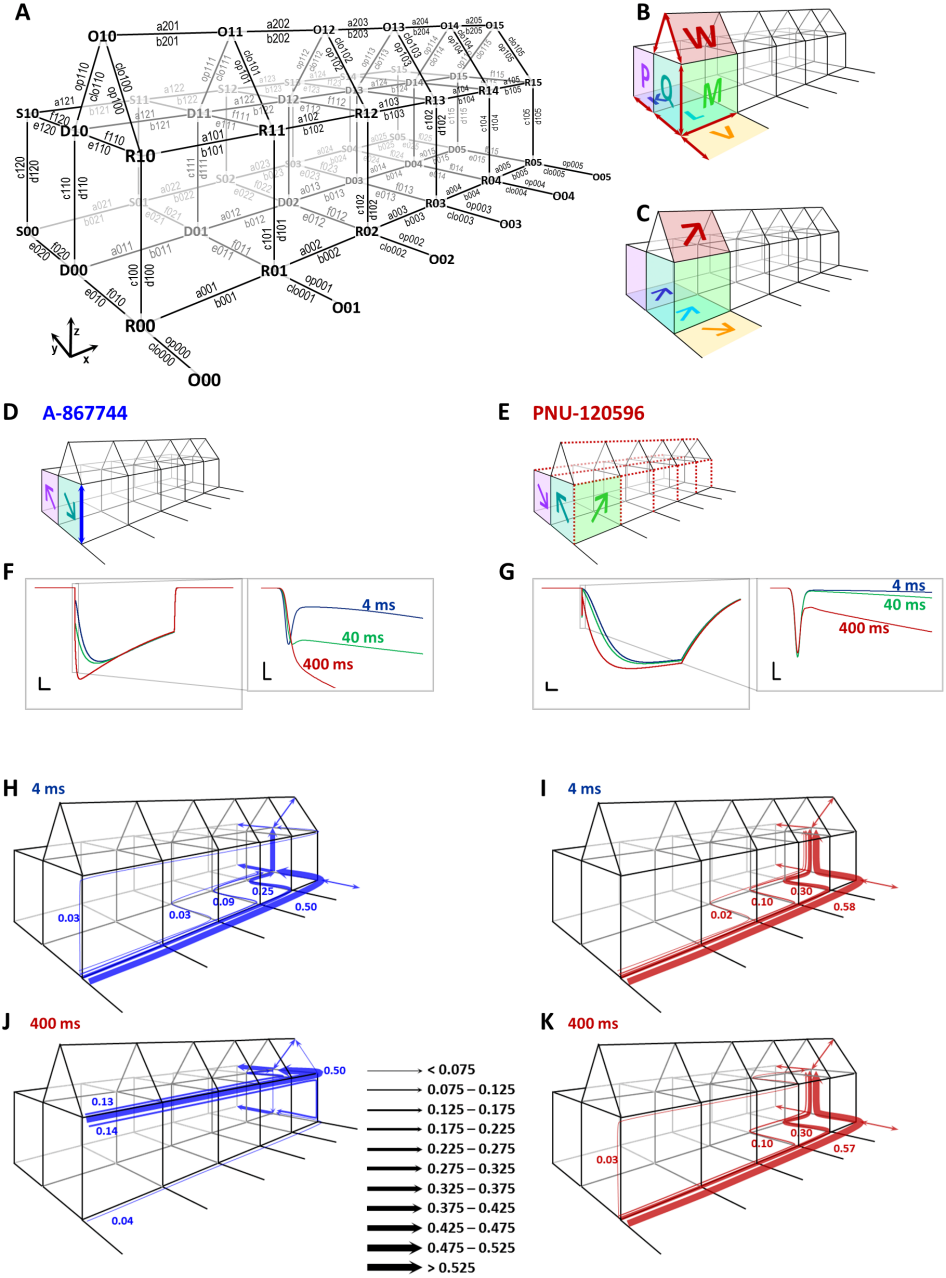
Kinetic simulations of modulator effects. (A) The model used in our simulations. States are denoted by three-character codes. The first character indicates the conformation: O (open), R (resting), D (desensitized), and S (slow desensitized). The second character indicates occupancy of the modulator binding sites (vacant “0” or occupied “1”), and the last character indicates the number of bound agonist molecules (0 to 5). Transitions are denoted by a four-character code (except opening and closing transitions) as shown in the figure. The first character, a letter, indicates the nature of transition: “a” and “b” are association and dissociation of agonist molecules, respectively; “c” and “d” are association and dissociation of the modulator, e, f, op (opening) and clo (closing) are conformational transitions with no binding/unbinding involved. The numbers indicate the location of individual transitions within the scheme along the three axes. The first digit indicates presence or absence of the modulator (*z* axis), the second digit indicates the conformation(s) in which, or between which the transition occurs (*y* axis), and the third digit indicates the agonist occupancy level (*x* axis). (B) A visual illustration of the construction of this Monod-Wyman-Changeux-type model. Red arrows indicate the 13 free rate constants, colored planes and large letters indicate allosteric factors. The same allosteric factors are effective at all agonist binding steps (see Table S1). The allosteric factors K, L, V, and W express the interaction between agonist binding and conformational transitions, P, and Q, between modulator binding and conformational transitions, and M expresses cooperativity between agonist and modulator binding. Although the free parameters discussed thus far fully determine all rate constants, it was convenient to introduce some additional factors called “symmetrical barrier factors”, because both forward and backward transitions are to be multiplied by them; in effect they modify the energy barrier of specific transitions. We introduced two such symmetrical barrier factors: zD modifies the rate of modulator association/dissociation (i.e., transitions along the *z* axis) to desensitized (D) states, while xRDOS modifies the rate of choline association (i.e., transitions along the *x* axis) to all modulator-bound states (R, D, O, and S). (C) Visual illustration of allosteric factors needed to reproduce modulator-free receptor behavior: agonist binding increased the propensity of the receptor to open (“V”), to desensitize (“L”), and to enter slow desensitized state (“K”), while all these conformational transitions increased agonist affinity. We also supposed that agonist binding will increase the probability of being open in the presence of both modulators (“W”). (D) Visual illustration of the major parameters required to qualitatively reproduce properties of A-867744-modulated currents. It was necessary to suppose an absence of cooperativity between agonist and modulator (“M” absent), high modulator association and dissociation rates (blue arrow), as well as a preference of resting and slow desensitized states. (E) Visual illustration of the major parameters required to qualitatively reproduce major properties of PNU-120596-modulated current. It was necessary to suppose hindered modulator accessibility of resting receptors (red dashed lines along the *z* axis), a cooperativity between agonist and modulator (“M”), hindered agonist accessibility of modulator-bound receptors (red dashed lines along the *x* axis), and a preference of desensitized state. (F) and (G) Simulated currents (sum of all open states) evoked by a 1 s coapplication of 10 mM choline and 1 µM of either A-867744 (F) or PNU-120596 (G). The three colors show the cases when different lengths of modulator pre-application preceded coapplication of the same modulator with choline. Scale bars: 0.01 (open fraction of the receptor population), 100 ms. Insets show initial phase on an expanded time scale: Scale bars: 0.01 (open fraction), 1 ms. (H) to (K) Net probability fluxes at the onset of currents after 4 (H and I) and 400 (J and K) ms preincubation by either A-867744 (H and J), or PNU-120596 (I and K). Text between J and K shows scaling of the arrows (fraction of the receptor population moved along a specific transition path during the 1 s pulse).

### Kinetic simulations

To provide a more intuitive understanding of the differences between the mechanisms of action of the two modulators, we performed kinetic simulations. We used a model that is similar in topology to one published earlier (Szabo et al., 2014). We defined resting (R), open (O), desensitized (D), and PAM-resistant slow-desensitized (S) states for both modulator-unbound and modulator-bound receptors (Fig. 13A). For the sake of simplicity, we only considered modulator-free (lower level) and modulator-bound (upper level) receptors, disregarding different degrees of occupancy of the five modulator binding sites. Figure 13A shows the denotation of the states and rate constants. States are denoted by a three-character code, where the first character indicates the conformation (Open, Resting, Desensitized or Slow desensitized; along the *y* axis), the second character indicates the occupancy of the modulator binding sites (vacant “0”, or occupied “1”; along the *z* axis), and the last character indicates the occupancy of the agonist binding sites (0 to 5; along the *x* axis). Transitions are denoted by a four-character code (except opening and closing transitions) according to their position along the three axes, as shown in the figure. While we allowed the opening of modulator-free receptors only from resting state (for justification see Pesti et al., 2014), modulator-bound receptors were allowed to open from both resting and desensitized states. The model had 48 states and 190 rate constants, out of which only 13 were free parameters (marked as red arrows in Fig. 13B). To calculate with the fewest possible number of free parameters, we used a Monod-Wyman-Changeux-type allosteric model. We defined seven allosteric factors (Fig. 13B) which determined the energetic interaction between agonist binding and opening for both the modulator-unbound (V), and the modulator-bound (W) receptor, agonist binding and desensitization (L), agonist binding and slow desensitization (K), agonist and modulator binding (M), modulator binding and desensitization (Q), and modulator binding and slow desensitization (P). Allosteric factors produce a shift of equilibrium between both pairs of parallel equilibria. This is illustrated by the wide arrows on the colored planes in Figs. 13C–13E. Arrows point to the state that was made energetically favorable by the allosteric factor. This may be done by accelerating the forward transition, decelerating the backward transition, or both. We defined two “balance factors” for each allosteric factor, which determined to what extent the forward and backward transitions are affected by the allosteric factor. Balance factors are denoted by two characters: the first (uppercase) letter indicates the allosteric factor they belong to, and the second (lowercase) letter indicates the axis along which they modify the rate constant. Balance factors could have a value between the reciprocal of the allosteric factor and the allosteric factor. For example, if *M* >1 (i.e., the association of the modulator increases the affinity of the agonist and vice versa), then *Mx* = 1∕*M* means that increased agonist affinity is caused by the decreased dissociation and an unchanged association rate of the agonist, while *Mz* = *M* means that the increased modulator affinity is solely due to the increased association rate. In addition, we introduced two symmetrical barrier factors to test hypotheses regarding binding site accessibility in a more straightforward manner. Symmetrical barrier factors elevate or lower the energy barrier between two specific sets of states. They did not interfere with detailed balance within the model because forward and backward rate constants were changed equally (see Table S1 for details). For all five subsequent agonist binding steps, we supposed the allosteric constants K, L, V, W and M to be constant. The values of the free parameters, allosteric, balance and symmetrical barrier factors, as well as the calculations and values of all transition rates are given in Table S1 for the simulated effects of both A-867744 and PNU-120596.

Agonist association was presumed to increase the tendency of receptors to open, to desensitize, and also to enter the slow desensitized state in the case of both modulators, as illustrated by the arrows representing allosteric factors K, L, V and W in Fig. 13E. To reproduce the main characteristics of the two modulators, however, we had to introduce significant differences in the allosteric constants M, P and Q, in the association and dissociation rates for the modulators and in the agonist association/dissociation rates for modulator-bound receptors (Figs. 13D and 13E).

We chose to simulate preincubation times of 4, 40 and 400 ms (Figs. 13F and 13G) because this range includes most of the changes caused by preincubation. We supposed that both modulators partition into the membrane phase and calculated their intramembrane concentration as described earlier (Szabo et al., 2014). Insets in these figures show the initial phase at expanded time scale, as shown for the experimental data. We aimed to qualitatively reproduce the major characteristics of the modulated currents, and not achieve a perfect fit of the simulated currents.

In the case of A-867744, we intended to examine what parameters are necessary to observe the exchange of the peak component for a slightly slower but augmented onset current, as well as the fast deactivation at the end of the agonist pulse. We found that the modulator association/dissociation rate had to be very high for both the resting and desensitized receptors (indicated by the blue arrow in Fig. 13D; for values see Table S1). However, there could be no significant cooperativity between agonist and modulator binding (*M* = 1): when we introduced even a small amount of cooperativity, the fast deactivation could not be preserved. The modulator had to have a higher affinity for the resting state of the receptor than to the desensitized conformation (*Q* < 1, indicated by the teal colored arrow), but this was compensated by a higher accessibility to the desensitized state (i.e., faster association/dissociation). We achieved this by introducing the symmetrical barrier factor zD (see Table S1). To reproduce the decay during the 1 s pulse of the agonist (coapplied with A-867744), we needed to suppose a somewhat higher affinity to slow the desensitized conformation (*P* > 1; purple arrow).

In the case of PNU-120596, we investigated the requirements for the unchanged peak component, slow onset and slow deactivation. Similar to our previous results, we found that slow deactivation required strong cooperativity between the agonist and modulator binding (*M* > 1; illustrated by the green arrow in Fig. 13E). In addition, we needed to suppose hindered accessibility of agonist binding sites of PNU-120596-bound receptors. We modeled this by using the symmetrical barrier factor xRDOS, as described earlier (Szabo et al., 2014). This is indicated by the red dashed lines in Fig. 13E. To reproduce the unchanged peak component and the delayed onset of the modulated current afterwards, we needed to define an extremely low modulator accessibility to resting state (i.e., the rate constants c100 to c105 and d100 to d105 were very low), as shown by the red dashed lines in Fig. 13E. In contrast to A-867744, we had to suppose that PNU-120596 preferred desensitized conformation (*Q* > 1, *P* < 1, see Fig. 13E).

To illustrate the effects of preincubation, net probability fluxes along decomposed main transition pathways were calculated as described in (Benndorf, Kusch & Schulz, 2012) (Figs 13H to 13K). Without sufficiently long preincubation (4 ms), receptors travelled similar pathways in the presence of both modulators (Figs 13H and 13I): agonist association was followed by desensitization (while a small fraction entered the modulator-free open state), and modulator association only occurred afterwards. After 400 ms of preincubation, however, the effect of the two modulators differed completely (Figs 13J and 13K). A-867744 readily associated to the agonist-free resting state receptors (accumulating in R10 in the absence of agonist), and upon agonist application, the majority of the receptors rapidly proceeded to the modulator-bound open state (O15), as well as to the other absorbing states (D05, D15, S05 and S15). In contrast, preincubation in PNU-120596 failed to produce a significant association to resting state, and the receptor population essentially travelled the same pathway as without preincubation.

## Discussion

The original classification of α7 nAChR PAMs distinguished compounds that predominantly affected the peak current amplitude (type I) and compounds that changed the gating characteristics of the receptors, causing prolonged activation instead of the unmodulated transient activity (type II) (Grønlien et al., 2007a). This classification has been suggested to be an oversimplification (Chatzidaki & Millar, 2015; Corradi & Bouzat, 2016) originating from insufficient time resolution of the recorded currents and a lack of detailed studies of mechanisms of action. Indeed, the archetypal type I PAMs 5-hydroxyindole and NS-1738 have been shown to prolong single channel open times and induce bursting, and they also produce a small but detectable prolonged current at the whole-cell level (Andersen et al., 2016). PAMs of α7 nAChR are prospective drugs for cognitive impairment, inflammation, acute and chronic neurodegenerative conditions, and different pain syndromes. Different mechanisms of action may be preferable for different therapeutic effects (Bouzat et al., 2018; Echeverria, Yarkov & Aliev, 2016). A-867744 is special because, on one hand, at prolonged, moderate increases in agonist concentration (such as in the case of increased choline levels around injured brain tissue) it would act as a type II PAM because it is able to reactivate desensitized receptors. On the other hand, when accurate timing is required to follow pulse-like elevations in agonist concentration, such as in the case of synaptic and perisynaptic α7 nAChRs, it would act as a type I PAM because it allows the receptors to deactivate as soon as the agonist concentration drops.

In this series of experiments, we intended to study the similarities and differences between two modulators. We found that the two modulators, A-867744 and PNU-120596, compete for the same binding site and can displace each other. This was evident from the fact that the presence of bound A-867744 to the receptor slowed the development of PNU-120596-mediated modulation more than 50-fold (compare Figs. 8 and 10). Similarly, A-867744 was unable to exert its effects on deactivation after the binding sites had been saturated with PNU-120596 (Fig. 12C). In addition, the displacement of A-867744 by PNU-120596 was clearly concentration-dependent (Figs. 5 and 6).

Both modulators seemed to have higher accessibility to desensitized conformation. However, in the case of A-867744 it was a mild difference: association to resting receptors occurred with a time constant of 169 to 332 ms, (depending on the exact experimental conditions (Figs. 1 and 2), and association to desensitized receptors occurred with a time constant of 47.8 ms when the binding site was (practically) unoccupied (Fig. 11). Simulations suggest that the binding reaction itself is much faster (otherwise the fast deactivation would not be possible), and the rate limiting step is the accumulation of the modulator in the membrane phase. On the other hand, in the case of PNU-120596, the desensitized state preference was extreme: association to desensitized state proceeded with a time constant of 97.8 ms (Fig. 8), while the time constant observed for resting state was 2,813 ms (Table 1, Fig. S3D), although the latter value comes from the parallel processes of accumulation within the membrane phase and association to both resting and desensitized states.

The most obvious difference between the two modulators is the radically different onset and offset times for their effects, which underlie their fundamentally different behavior during short agonist pulses. This is based on the duration of single channel open and closed times. In the presence of agonist alone, α7 nAChRs almost exclusively open for durations less than a millisecond, and approximately half of the openings were found to be <100 µs (DaCosta et al., 2011). In the presence of PNU-120596, openings are radically prolonged; the open time histograms consisted of multiple components, the most typical form of openings was several second-long bursts of opening separated by long closed times (DaCosta et al., 2011; Williams, Wang & Papke, 2011b). It is obvious that the onset and offset rates are determined by single channel dwell times: it is impossible to produce faster onset and offset on the macroscopic level than the dominant single channel dwell times. Onset rates in the presence of PNU-120596 were considerably faster than the steady-state dwell times, but during the onset, PNU-120596 probably gradually occupies the binding sites while the single channel dwell times are progressively prolonged. All other positive modulators studied at the single channel level thus far caused a prolongation of open times, although most exerted this effect to a much lesser extent (Andersen et al., 2016). In the case of A-867744, no single channel analysis has thus far been published, but based on the macroscopic currents, mean open and closed times cannot be higher than ∼1 ms. This means that this compound acts predominantly by increasing the probability of opening, not by introducing an energetically stable open conformation.

Throughout this study, we attempted to infer modulator binding site occupancy from the observable effects of the modulator. One important conclusion from the results of this study is that this must be done with caution. A modulator can alter certain properties of the current with great efficacy, while other properties rather inefficiently. For example, as shown in Fig. 12C, during the approximately 1 s long deactivation after choline + PNU-120596 coapplication, A-867744 was unable to alter the rate of decay. We cannot conclude that A-867744 was unable to bind since after only 200 ms of A-867744 perfusion it had already radically altered the onset phase, the current itself, and the decay after 1 s of repeated choline + PNU-120596 coapplication.

One explanation for this result might be that during deactivation, we can only see the current on the fraction of receptors that still bind four to five PNU-120596 molecules (occupancy of this many binding sites is required for the modulation to be effective) (DaCosta & Sine, 2013). For this reason, A-867744 molecules are unable to bind to open receptors, and they can only associate to those receptors that have already been deactivated. Deactivated receptors remain silent until the next pulse of agonist. For A-867744, no such stoichiometric requirement is known thus far; in fact, we find it likely that partial occupancy (1 or 2 binding sites per receptor) may be enough to produce the fast, augmented onset as well as the fast offset, which are characteristics of A-867744-mediated modulation. This would explain why at the same concentration, A-867744 seemed to be more efficient (see Figs. 5 or 12C), while it is not known to have higher affinity (∼1 µM) (Malysz et al., 2009) than PNU-120596 (216 nM) (Hurst et al., 2005).

## Conclusions

We found significant differences between the mechanisms of action of two type II PAMs. Most importantly, PNU-120596 has a very limited ability to bind to resting receptors, while A-867744 can readily associate to this conformation. In addition, PNU-120596 is able to induce prolonged bursts of openings, while A-867744 acts by destabilizing desensitized state rather than stabilizing an open conformation. Kinetic simulations suggest that the association and dissociation of A-867744 to resting receptors must be faster by several orders of magnitude, although the affinity of the two compounds is similar. In addition, while the effects of PNU-120596 could only be reproduced by supposing cooperativity between agonist and modulator binding, no such interaction was predicted for A-867744. These differences result in completely different behaviors under short agonist pulses. We demonstrated that the effects of type II PAMs under physiological spatiotemporal patterns of agonist concentration can be widely different, and therefore it is desirable to perform a more detailed investigation of the mechanisms of action when PAMs of the α7 nAChR are developed as prospective drugs. The special properties of A-867744 seem advantageous from a therapeutic point of view: it behaves as a type II PAM at prolonged increases of agonist concentration, while it is able to follow brief pulses of agonist release with millisecond precision as a type I PAM. To produce this type of modulation, the key properties are a moderate prolongation of open time and the ability to freely associate to resting conformation.

##  Supplemental Information

10.7717/peerj.7542/supp-1 Table S1 Parameters of kinetic simulations All free and calculated parameters for modeling the effects of both A-867744 and PNU-120596. Rate constants are in ms^−1^ units, with the exception of association rates (a001 to a215), which are in s^−1^μM^−1^ units. Free parameters are in shaded cells. We needed 13 free rate constants, and 7 allosteric factors. For each allosteric factor we defined two barrier factors, which determined to what extent forward and backward transitions are affected by the allosteric factor. Barrier factors are denoted by two characters, the first (uppercase) letter indicates the allosteric factor they belong to, and the second (lowercase) letter the axis along which they modify rate constants.

10.7717/peerj.7542/supp-2 Supplemental Information 1 Amplitude, net charge flux, and time constant data from individual experiments These figures serve to complement Figs. 1 to 7, with data regarding pre-incubation duration dependence of: (i) relative amplitude of the initial component and (ii) the main component, (iii) relative net charge flux (AUC), (iv) time constants of onset and (v) deactivation, whichever is relevant in that particular experiment. Thin lines show data from individual cells, thick dashed lines indicate arithmetic mean (for amplitudes and AUC values), and geometric mean (for time constants).

10.7717/peerj.7542/supp-3 Supplemental Information 2 Properties of currents recorded from individual cells Summary of all experiments with the “*PRE*”, “*CO*”, and “*POST*” protocols. Current traces from individual cells are shown, as well as the major parameters obtained from these currents.

## Additional Information and Declarations

### Data Availability

The following information was supplied regarding data availability: Raw data are available as a Supplemental File.
