# Supplementary material for: Type I-like behavior of the type II α7 nicotinic acetylcholine receptor positive allosteric modulator A-867744
Source: PeerJ. 2019 Sep 2;7:e7542. doi: 10.7717/peerj.7542 (PMC6727837; doi:10.7717/peerj.7542)
Supplement: Supplemental Information 1 — These figures serve to complement Figs. 1 to 7, with data regarding pre-incubation duration dependence of: (i) relative amplitude of the initial component and (ii) the main component, (iii) relative net charge flux (AUC), (iv) time constants of onset and (v) deactivation, whichever is relevant in that particular experiment. Thin lines show data from individual cells, thick dashed lines indicate arithmetic mean (for amplitudes and AUC values), and geometric mean (for time constants). [file peerj-07-7542-s002.pdf]

A-867744 → choline

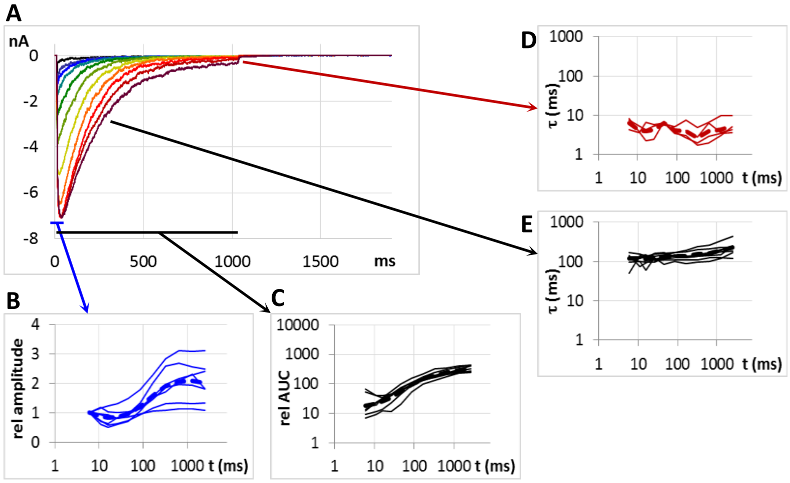

Figure S1

A-867744 → A-867744 + choline

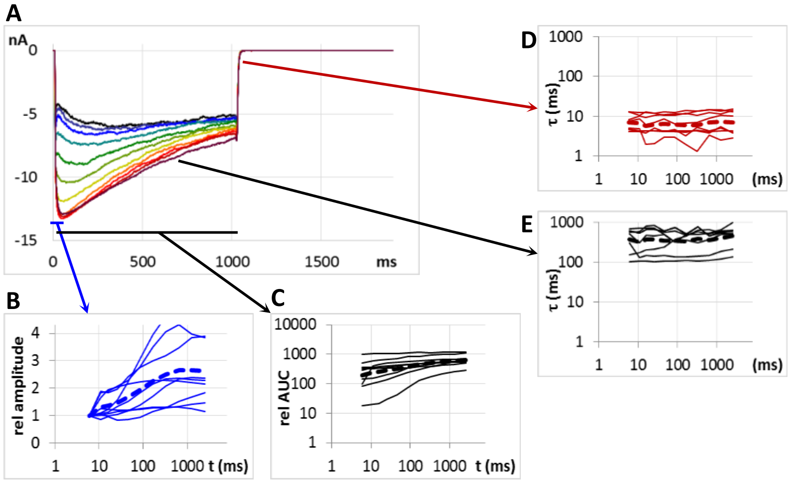

Figure S2

PNU-120596 → choline

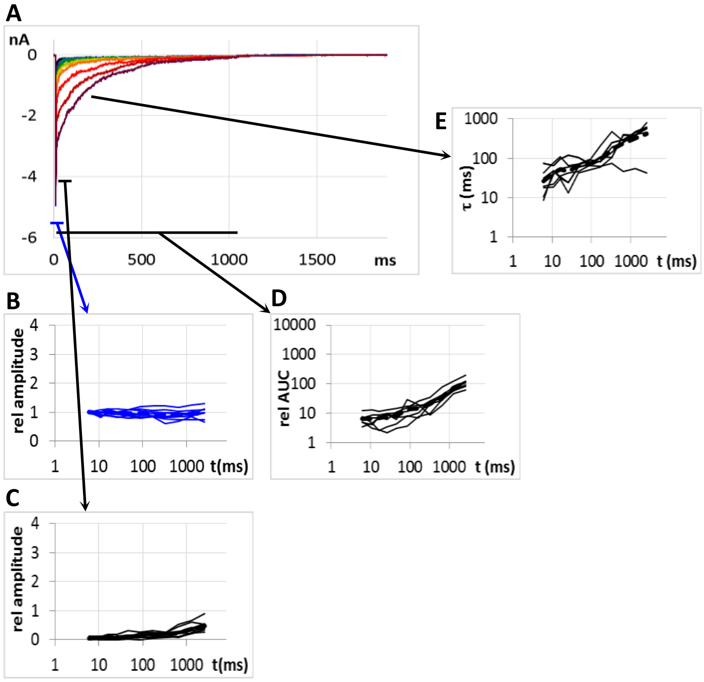

Figure S3

PNU-120596 → PNU-120596 + choline

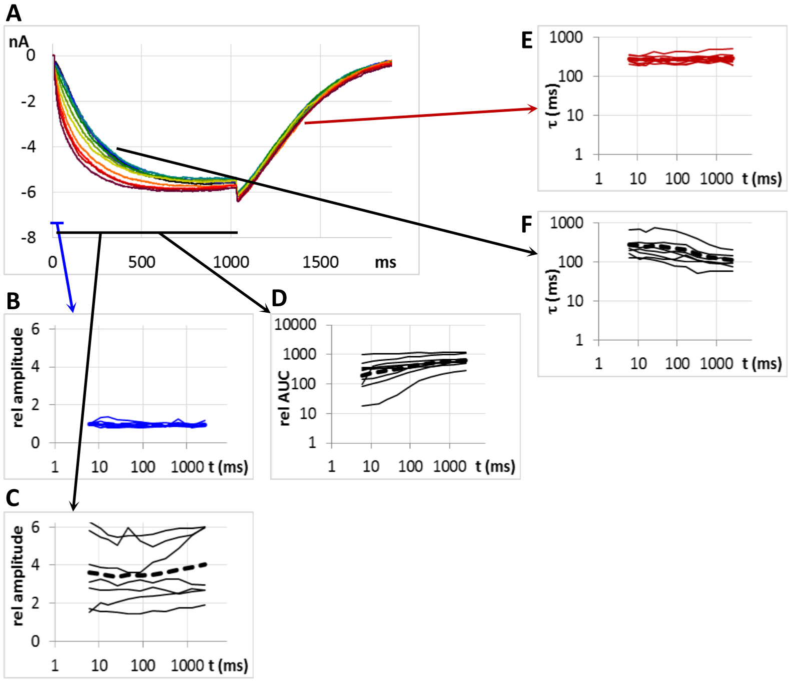

Figure S4

A-867744 → PNU-120596 + choline

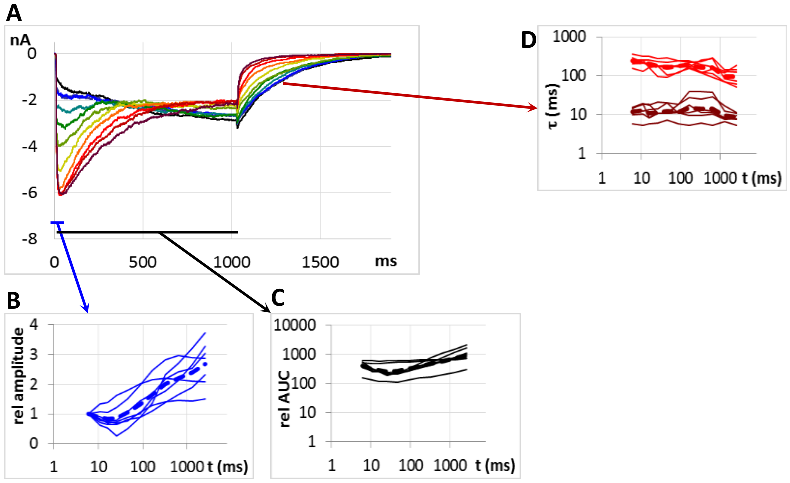

Figure S5

A-867744 → 10 μM PNU-120596 + choline

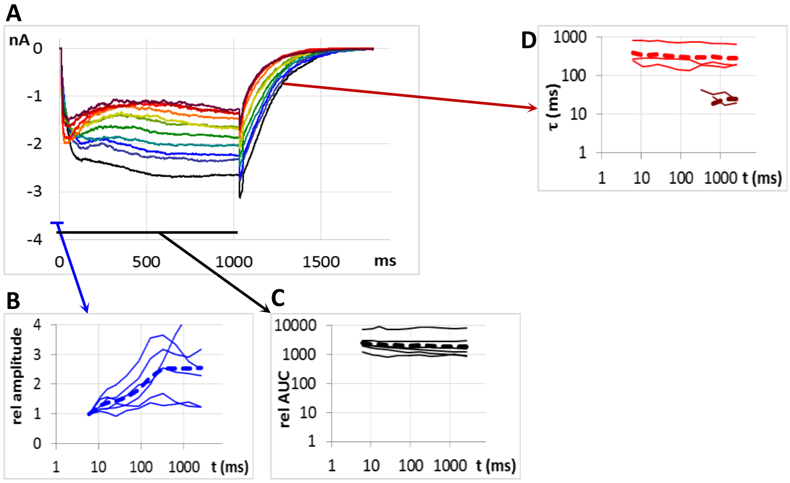

Figure S6

PNU-120596 → A-867744 + choline

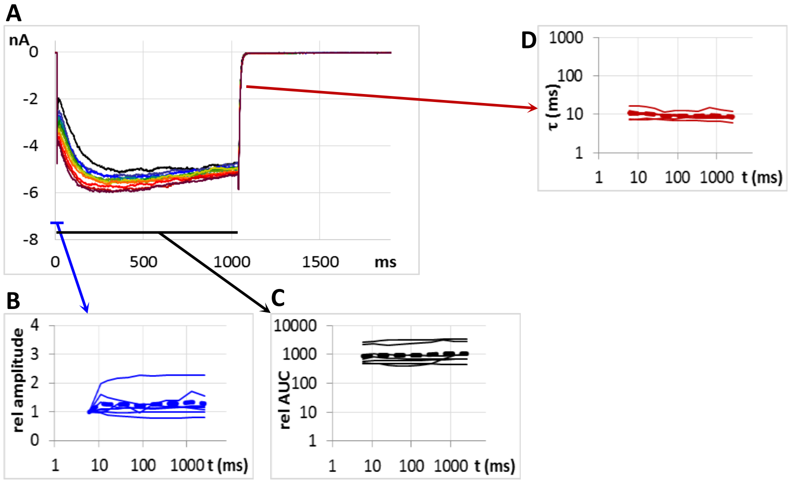

Figure S7
